# Reading the Mind in the Eyes Test Scores Demonstrate Poor Structural Properties in Nine Large Non-Clinical Samples

**DOI:** 10.1177/10731911251328604

**Published:** 2025-03-29

**Authors:** Wendy C. Higgins, Victoria Savalei, Vince Polito, Robert M. Ross

**Affiliations:** 1 Macquarie University, Sydney, NSW, Australia; 2 University of Melbourne, VIC, Australia; 3 University of British Colombia, Vancouver, BC, Canada

**Keywords:** factor analysis, Reading the Mind in the Eyes Test, validity, reliability, social cognition

## Abstract

The Reading the Mind in the Eyes Test (RMET) is widely used in clinical and non-clinical research. However, the structural properties of RMET scores have yet to be rigorously examined. We analyzed the structural properties of RMET scores in nine existing datasets comprising non-clinical samples ranging from 558 to 9,267 (median = 1,112) participants each. We used confirmatory factor analysis to assess two theoretically derived factor models, exploratory factor analysis to identify possible alternative factor models, and reliability estimates to assess internal consistency. Neither of the theoretically derived models was a good fit for any of the nine datasets, and we were unable to identify any better fitting multidimensional models. Internal consistency metrics were acceptable in six of the nine datasets, but these metrics are difficult to interpret given the uncertain factor structures. Our findings contribute to a growing body of evidence questioning the reliability and validity of RMET scores.

The Reading the Mind in the Eyes Test (RMET; Baron-Cohen et al., 2001) is one of the most widely used measures of social cognitive ability. It includes 36 items, each of which comprises a black and white photograph of a person’s eyes and four mental state descriptors (see Figure 1). The task is to select which of the four mental state descriptors best describes what the person in the photograph is thinking or feeling. Under the original coding scheme, a single sum score from 0 to 36 is calculated based on the total number of correct responses, with higher scores indicating greater social cognitive ability. Additionally, at least 170 studies have calculated subscores based on the valence of the test items (that is, “positive,” “negative,” or “neutral”; Higgins et al., 2024).

**Figure 1.**
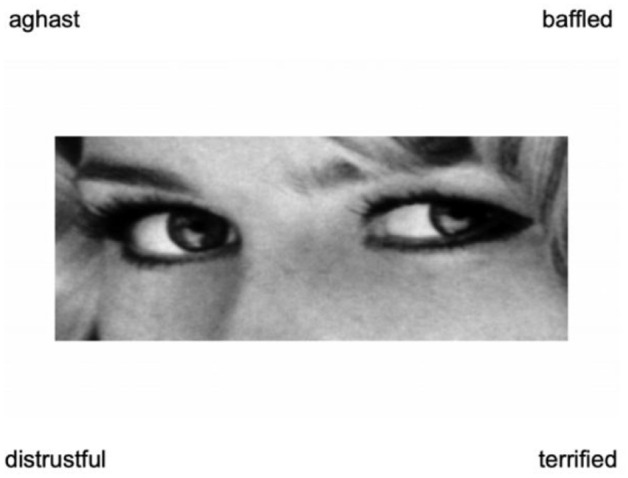
Sample RMET Item. *Note*. The target response for this item is “distrustful.” The RMET is freely available from https://www.autismresearchcentre.com/tests/. RMET = Reading the Mind in the Eyes Test.

The RMET has been used in more than 1,500 empirical studies, translated into at least 39 different languages, and administered to samples from more than 75 different clinical populations (Higgins et al., 2024). The RMET is also endorsed by the United States’ National Institute of Mental Health’s Research Domain Criteria Initiative as a “current best option” for assessing the ability to understand mental states. Nonetheless, substantial concerns have been raised about the validity of RMET scores (Higgins et al., 2024, 2025; Higgins, Ross, et al., 2023; Higgins, Savalei, et al., 2023; Johnston et al., 2008; Oakley et al., 2016; Olderbak et al., 2015; Silverman, 2022; cf., Murphy & Hall, 2024).

In the most comprehensive examination of the validity of RMET scores to date, Higgins et al. (2024) conducted a systematic scoping review of quantitative validity evidence for RMET scores across 1,461 studies. Six categories of validity evidence were coded (internal consistency, test-retest reliability, factor structure, known group validity, convergent validity, and discriminant validity), revealing two striking findings. First, validity evidence for RMET scores was infrequently reported, with only 37% of studies reporting evidence from at least one of the six coded categories of evidence. Second, when validity evidence was reported, results frequently indicated low levels of validity.

The lack of validity evidence reported in the RMET literature stands in stark contrast to best practice guidelines from the American Psychological Association, which state that, where possible, validity evidence should be provided in every empirical study and should be based on the data analyzed within a given study (i.e., evidence should be sample-specific; Appelbaum et al., 2018). Sample-specific evidence is important because the properties of test scores, including factor structure and internal consistency, can vary across samples. Therefore, without sample-specific validity evidence, consumers of research studies lack crucial information needed to evaluate the validity of test scores or research findings based on those scores (Flake & Fried, 2020; Flake et al., 2017), with serious implications for the interpretability of the vast RMET literature.

The structural properties of RMET scores are of particular concern because only 13 (0.01%) of the 1,461 studies surveyed by Higgins et al. (2024) examined the factor structure of RMET scores, and all 13 of them failed to report key information required to assess model fit, reported evidence of unacceptable model fit for tested models, or both (see Higgins et al., 2024 for details). While internal consistency metrics were reported more frequently, with 157 (10.7%) of the surveyed studies reporting a measure of internal consistency, the paucity of evidence for acceptable model fit impacts the interpretability of these internal consistency metrics because knowledge of the factor structure of test scores is necessary to correctly calculate and interpret internal consistency metrics.^1^ Moreover, only 120 (40%) of the 201^2^ reported estimates of internal consistency met conventionally accepted minimal thresholds.

The lack of information about the factor structure and internal consistency of RMET scores constitutes a substantial gap in the validity evidence for RMET scores. This is because RMET sum scores are almost always interpreted as measurements of social cognitive ability (Higgins et al., 2024), and interpreting sum scores as a measurement of a specific psychological attribute requires evidence that the scores are unidimensional and reliable (McNeish, 2024; Slaney, 2017; Widaman & Revelle, 2023).^3^ Information about the structural properties of RMET scores is also critical when interpreting external sources of validity evidence that are based on RMET sum scores, such as known group, convergent, and discriminant validity evidence, and the structural properties of RMET scores should be assessed prior to interpreting these types of validity evidence (Slaney & Maraun, 2008).

Concerns have also been raised about the way in which the “correct” responses for RMET items were established (Higgins et al., 2024, 2025; Johnston et al., 2008; Kim et al., 2024). The RMET photographs were taken from magazines, and the associated mental state descriptors were generated by the test’s creators (i.e., Baron-Cohen et al., 2001) and then validated in small groups (for a more detailed critique of the test construction process for the RMET see Higgins et al., 2024). Forty candidate RMET test items (and their “correct” responses) were then administered to a group of 225 participants to verify that the following two consensus criteria were met: (a) at least 50% of participants selected the “correct” mental state descriptor and (b) no more than 25% of participants selected the same “incorrect” mental state descriptor. The 36 items that met both criteria make up the RMET.

Given that the “correct” RMET responses were verified via consensus in a single study conducted using convenience samples in the United Kingdom, it is open to question whether the target responses should be considered correct in samples where these consensus criteria are not met (Higgins et al., 2024). Item-level data have rarely been reported in subsequent studies; moreover, where reported, a subset of items frequently failed to pass at least one of the two consensus criteria from the original study (Higgins et al., 2024; Higgins, Ross, Langdon, et al., 2023). In fact, in one study conducted in China, an “incorrect” response was recoded as the “correct” response because it was selected by more than 60% of participants, while less than 30% of participants selected the target response (Zhang et al., 2016). Issues such as these suggest that the “correct” responses do not always generalize across samples, making it important for studies to examine the distribution of responses to individual items before interpreting RMET scores.

In the current paper, we examine the structural properties of the RMET in nine large, pre-existing datasets, with a focus on evaluating the factor structure and internal consistency of RMET scores. While these nine datasets are all drawn from previously published studies, factor analyses using these datasets have not been published, and internal reliability metrics have only been reported for three of them. For each dataset, we perform five analyses on RMET data. First, using confirmatory factor analysis (CFA), we test a single-factor model because the RMET was designed to be a unidimensional test that produces a single sum score (Baron-Cohen et al., 2001). Second, again using CFA, we assess a three-factor model based on the valence subscales proposed by Harkness et al. (2005), which is the most widely used, theoretically derived model for generating RMET subscores (Hudson et al., 2020). Third, using exploratory factor analysis (EFA), we attempt to identify alternative data-driven factor models that might fit the data better than the theoretically derived models. Fourth, using alpha and omega reliability estimates, we examine the internal consistency of RMET scores. Fifth, for each RMET item, we calculate the percentage of participants that selected each of the four response options and compare these percentages with the consensus criteria set out by Baron-Cohen et al. (2001).

## Method

### Transparency and Openness

This project, including our plan for choosing which datasets to re-analyze and how we would re-analyze them, was pre-registered (https://osf.io/5dx8t). Since the time of pre-registration, the project acquired an additional collaborator, Victoria Savalei, who brought statistical expertise to the project. In addition, we read more deeply on best practices for conducting factor analysis and computing internal consistency estimates. As a result, we have made several deviations from the pre-registration to ensure that all analyses are consistent with current best practices. We clearly identify all deviations as they are introduced and provide justifications for each of them.

Using data from Higgins et al.’s (2024) scoping review, we identified all studies that met our inclusion criteria of administering the unabbreviated, English language version of the RMET to a non-clinical sample. Following our pre-registration, we contacted the authors of the 15 studies with the largest sample sizes that met these criteria (these studies are demarcated by an asterisk [*] in the reference list).^4^ In two cases, we were unable to make contact with the author in possession of the data (De Paoli et al., 2020; Weisman et al., 2015), and one research group declined to share their data due to the ongoing nature of their research project (Carroll et al., 2021). Additionally, a subset of the identified studies used overlapping datasets (Dodell-Feder et al., 2020; Germine et al., 2015; Hartshorne & Germine, 2015; Pearce et al., 2019a, 2019b), and in these instances, we analyzed the version of the dataset used in the most recently published study. In total, we analyzed nine independent datasets, each of which we refer to using the first author’s last name. Anonymized data and analysis code are available via the project’s OSF page (https://osf.io/cyt6m/).^5^

### Participants

Table 1 reports properties of each dataset, including summary statistics for participants’ demographic information and sample sizes. Participants were recruited at science festivals and a museum (Pearce et al., 2019b); via the online participant pool platforms MTurk (Almaatouq et al., 2021; Floyd & Woo, 2020; Panero et al., 2016) and TurkPrime (Schmalor & Heine, 2021); via university student participant pools (Nahal et al., 2021; Vonk et al., 2015); and via self-selection through the Testmybrain.org website (Dodell-Feder et al., 2020) and a link in a New York Times article (Kidd & Castano, 2017).

**Table 1:** Original Study and Sample Characteristics.

Study	Sample size—original study	Sample size—current analyses	Demographics—original study	Age mean (range)—original study	Recruitment method	Data collection method	Compensation	Attention check/s	Specific response to each item available^a^	Reported reliability estimate—original study
Almaatouq et al. (2021)	1,200	1,181	— 56% Women	—	MTurk	Online via MTurk	Up to US $17.00	Yes	Yes	None
Dodell-Feder et al. (2020)	9,271	9,267	84% White 63% Women	30 (10–70)^b^	TestmyBrain.org	Online via TestmyBrain.org	No	No	Yes	Alpha = .71
Floyd and Woo (2020)	1,118	1,016	75% White 47% Women	39 (18–74)	MTurk Master Workers	Online via MTurk	US $2.50	Yes	Yes	None
Kidd and Castano (2017)	1,243	1,112	83% White 72% Women	44	Link in a New York Times article	Online	No	No	Yes	Alpha = .56
Nahal et al. (2021)	1,168	1,168	— 62% Women	19 (17–54)	University student participant pool in Canada	Pen and paper test	Unclear	No	No	None
Panero et al. (2016)	792	558	— 51% Women	35 (18–76)	MTurk	Online via MTurk	US $2.00	No^c^	Yes	None
Pearce et al. (2019b)	757	709	100% White 56% Women	Men: 43 (18–75) Women: 38 (18–74)	Science festivals and a museum in the UK	An iPad on location	Unclear	No	No	None
Schmalor & Heine (2021)	972	972	74% White 62% Women	37	TurkPrime	Online via TurkPrime	Unclear	Yes	Yes	Alpha = .73
Vonk et al (2015)	901	868	76% White 82% Women	20	University student participant pool in United States	Online via Survey Monkey	Course credit	No	No	None

a This column indicates whether datasets coded which of the four possible responses were selected for each RMET item, or whether responses were only coded as correct/incorrect.

b This age range refers to a larger dataset, and the study does not report the mean age or age range specific to the RMET.

c While no attention checks were included, participants who took too much or too little time reading a passage of text required for the survey were excluded as this was taken to indicate that they had not read the passage.

Sample sizes for the nine datasets as analyzed in the original studies ranged from 757 to 9,271 (median = 1,118). With the exception of the Floyd dataset, the majority of participants in each dataset were women (range 47%–82%, median = 62%). In the six studies that reported ethnicity, participants were predominantly White (range 74%–100%, median = 80%). Mean age ranged from 19 to 44 (median = 37). In preparing the datasets for analysis, we applied the same exclusion criteria as the original studies with the following exceptions: as per our pre-registration, we excluded from our analyses any participants who did not provide a response to all 36 RMET items. The number of participants excluded for missing RMET data ranged from 0 to 131 (median = 19). We also excluded the data associated one participant ID in the Dodell-Feder dataset because the participant ID appeared four times. After participant exclusions, sample sizes used for analysis ranged from 558^6^ to 9,267 (median = 1,016).

### Measures

All studies administered the 36-item RMET in its original English language format. None of the studies conducted factor analysis on RMET scores, while three studies reported alpha as a measure of internal consistency (see Table 1).^7^

### Statistical Analysis

Statistical analyses were run in R (version, 4.3.1.; R Core Team, 2022) using RStudio (version 2023.9.1.494; Posit Team, 2023).

We calculated item-level RMET responses for each dataset. The specific response to each item (as opposed to correct/incorrect coding) was available for six of the nine datasets (see Table 1), and in these instances, we calculated the percentage of participants who selected each of the four response options. For the three datasets for which this information was not available, we were only able to calculate the percentage of participants selecting the correct response. We compared response percentages with the criteria set out in Baron-Cohen et al. (2001) for items to be included in the final version of the RMET: at least 50% of participants select the target response, and no more than 25% of participants select the same incorrect response.

We assessed the suitability of each dataset for factor analysis via the Kaiser–Meyer–Olkin measures of sampling adequacy (MSA) test, the Bartlett’s test of sphericity, and the test of the independence model. We calculated the MSA and Bartlett’s test using the FACTORABILITY function in the *EFA.dimensions* package (version 1.7.9; O’Connor, 2023). Because the test uses binary scoring (i.e., each item is scored as correct or incorrect), we specified the use of a tetrachoric correlation matrix. MSA values range from 0 to 1, with higher values indicating increased suitability of the data for factor analysis. A value less than .50 is considered “unacceptable,” while values above .80 are considered “meritorious” (Hair, 2019; Kaiser, 1974). Bartlett’s test assesses the null hypothesis that the correlation matrix does not differ from the identity matrix, and this null hypothesis should be rejected (Bartlett, 1954; Hair et al., 2010). While not pre-registered, we also report the test of the independence model, which was calculated using the *lavaan* package (version 6.16; Rosseel, 2012). The test of the independence model assesses the same null hypothesis as Bartlett’s test but using the correct sampling distribution for the matrix of tetrachoric correlations, and thus, it will be more accurate than the result from the EFA.dimensions package.^8^

We conducted EFA and CFA using the *efa* and *cfa* functions in the *lavaan* package.^9^ Because the data are binary, we specified all indicators as ordered categorical (in *lavaan*, ordered = TRUE), which triggers the diagonally weighted least squares estimator with robust standard errors and mean and variance adjusted test statistic (i.e., estimator = “WLSMV”). We tested two theoretically derived CFA models (i.e., single-factor and three-factor valence-based models). We report the results of these CFA models analyzed within the full sample for each dataset. This is a deviation from the pre-registration where we specified that all models would be tested using half of each dataset. However, these two CFA models are both theoretically derived, and no data-driven modifications would be performed. As such, we realized in retrospect that there is no statistical reason not to run these two CFA analyses in the full datasets, and doing so would allow us to obtain more stable estimates. In contrast, for the EFA analyses, we split each dataset in half so that we could conduct EFA in one half of each dataset and then conduct additional CFAs in the other half if we identified promising statistically derived factor models.

For the EFA analyses, datasets were divided using the *data.partition* function in the *datawizard* package (version 9.0; Patil et al., 2022). We used the *scree* function in the *psych* package (version 2.3.9; Revelle, 2023) to plot the eigenvalues of the tetrachoric correlation matrix in each dataset, and based our decisions about the number of factors to extract primarily on the visual inspection of the resulting scree plots.^10^ Specifically, we looked for an “elbow” in each scree plot, where the distance between neighboring eigenvalues becomes dramatically smaller, and retained the number of factors above the elbow. Because a single-factor EFA model is equivalent to a single-factor CFA model, we did not run EFA for single-factor models. Instead, we conducted EFA analyses on datasets where scree plots suggested more than one factor. In these datasets, we extracted the number of factors suggested by the scree plot. While not pre-registered, we also always considered a three-factor model in these datasets because this dimensionality aligns with the practice of calculating valence-based subscores. For all EFA analyses, we used geomin rotation with *lavaan*’s default settings. In supplementary materials, we also report factor loadings based on oblimin rotation because different rotation methods can produce more interpretable factor loading patterns.

We evaluated CFA models based on model fit indices (described below), the matrix of residual correlations, and factor loadings. We evaluated EFA models based on factor correlations, factor loadings, communalities, simplicity of the structure (where the simplest structure is defined as the structure with the least number of items that load onto more than one factor), the conceptual fit of the model based on theoretical considerations related to social cognition, and model fit indices. When considering model fit indices in the context of EFA, it is important to note that good fit is a necessary, but not sufficient, condition for accepting an EFA model. Unlike CFA, where the model specifies which items are to load onto which factors, EFA allows all items to load onto all factors. As such, EFA models can fit the data better even when they do not result in an interpretable pattern of factor loadings. This makes it critical to consider the theoretical appropriateness and interpretability of EFA models. For both CFA and EFA, if a model did not converge, it was taken as evidence that the model was not an appropriate representation of the data, and the model was rejected.

Following best practices (Appelbaum et al., 2018; Kline, 2023), we report the chi-square test of exact fit; however, due to the sensitivity of the chi-square test to sample size (Bergh, 2015; Kline, 2023), we base model evaluation primarily on the following approximate fit indices: the comparative fit index (CFI), the Tucker–Lewis index (TLI), the root mean square of residuals (SRMR), and the root-mean-square error of approximation (RMSEA).^11^ CFI and TLI are relative fit indices, which compare model fit to an independence model. Higher values indicate better model fit (see Table 2 for traditional cutoff values for each fit index).^12^ In contrast, SRMR and RMSEA are absolute fit measures, which compare the correlation matrix from the observed data with the correlation matrix implied by the factor model (Bandalos & Finney, 2018). Lower values indicate better model fit for both SRMR and RMSEA. We assessed fit indices against the cutoff values outlined in Table 2; however, we note that the use of fixed cutoffs can be problematic, making it important to also consider the factor loadings, residual correlations, and theoretical support for each model (Kline, 2023; Marsh et al., 2004; McNeish & Wolf, 2023; Stanton et al., 2023). For all models, we also made attempts to identify the largest sources of the model misfit by examining the largest residual correlations, with residuals >.20 taken as indicators of possible local misfit of the model.

**Table 2:** Traditional Cutoff Values for Fit Indices.

Fit index	Category of index	Traditional cutoff
Chi-square (χ^2^) test of exact fit	Exact	*p* > .05
Comparative fit index	Relative	>.95
Tucker–Lewis index	Relative	>.95
Root-mean-square error of approximation	Absolute	<.08 suggests acceptable fit <.05 suggests good fit
Root mean square of residuals	Absolute	<.08

*Note*. Traditional cutoffs are taken from Bandalos and Finney (2018) and Kline (2023).

*Lavaan* prints both weighted least square mean and variance adjusted (WLSMV)-based fit indices (denoted by “scaled” in lavaan output), which are commonly reported for categorical data, as well as the new normal-theory maximum-likelihood-based fit indices (ML; denoted as “robust” in *lavaan* output), recommended by Savalei (2021). Where available in the *lavaan* output, we include both sets of indices in tables; however, we base our model assessments on the ML-based indices where available. ML-based indices tend to be more accurate for binary data because they approximate what the fit index value would have been had the data been continuous, allowing for reference against the commonly used ML fit index cutoffs (see Table 2), while WLSMV fit indices have different population values and tend to overestimate model fit for categorical data when judged against traditional cutoffs (Savalei, 2021). A disadvantage of the ML-based fit indices is that they cannot be obtained (or they will produce poor results) when the input correlation matrix is not positive definite, which can happen with categorical data. Unless otherwise specified, in the main test, we refer to the ML-based fit indices.

Factor loadings of at least 0.30 are considered acceptable for interpreting the structure of an EFA model (Hair, 2019). However, a loading of 0.30 means that the factor only accounts for approximately 10% of the variability in the indicator (i.e., RMET item). As such, Hair (2019) suggested that to be “practically significant” (p. 151), loadings should be at least 0.50, meaning that the factor accounts for approximately 25% of variance in the indicator. Because our primary aim in this paper is to identify the factor structure of RMET scores, for EFA models, items were considered to load onto a particular factor if the corresponding loading was at least 0.30. In cases where an item had loadings of at least 0.30 on multiple factors, the item was interpreted as loading onto the factor with the highest loading, unless there was a clear theoretical motivation to favor the weaker loading or to allow cross loadings. For CFA models, factor loadings were also deemed acceptable if they were at least 0.30. For EFA models, we also report the communalities for each item, which is the proportion of each item’s variance that can be explained by the extracted factors. Values range from 0 to 1, and the higher the value, the more of the item’s variance can be explained by the factors.

To assess the internal consistency of RMET scores based on a single-factor model, we calculated alpha based on the tetrachoric correlation matrix (which estimates the reliability of a hypothetical total score obtained by summing the underlying continuous items) and a categorical version of omega, which gives the reliability of the total score obtained by summing categorical items (rather than a hypothetical total score obtained by summing the underlying continuous items; Green & Yang, 2009). These versions of alpha and omega were calculated using the *compRelSEM* function in the *semTools* package (version 5-6; Jorgensen et al., 2022), using the *lavaan* object representing our specified single-factor CFA model as input.^13^ Alpha and omega values above .70 were interpreted as the minimum acceptable levels of internal consistency, with values above .80 being preferrable (Clark & Watson, 2019). Critically, the interpretation of alpha and omega relies on knowledge of the factor structure of the data. In particular, alpha assumes unidimensionality and approximately equal factor loadings for each item (Flora, 2020). Omega reliability estimates can be computed based on any factor model; however, they are only meaningful when computed based on models that are appropriate for the data (Savalei & Reise, 2019) and, thus, were only calculated if an appropriate multidimensional model was identified.

We also calculate the mean interitem correlation for each dataset. This statistic is important to consider when evaluating factor structure and internal consistency because fit indices, factor loadings, alpha, and omega are all sensitive to the strength of interitem correlations. It has been shown that the chi-square test rapidly loses power with lower correlations among the variables, and therefore absolute indices of fit (SRMR, RMSEA) will have lower sensitivity to misspecification when correlations are low as well (Miles & Shevlin, 2007; Saris et al., 2009; Savalei, 2012; Steiger, 2000). CFI and TLI have also been shown to be affected by interitem correlations (Miles & Shevlin, 2007). Because reliability indices are functions of interitem correlations, a lower mean interitem correlation will also result in lower values for alpha and omega. For a unidimensional model, a mean interitem correlation of less than .15 is considered low (Clark & Watson, 2019).

## Results

Mean RMET scores for each dataset ranged from 24.5 to 27.7 (median = 26.5; see Table S1). All datasets met the cutoff of at least 0.5 for the Keiser–Meyer–Olkin test, and the null hypothesis that the variables are uncorrelated was rejected by both Bartlett’s test as implemented in the *EFA.dimensions* package and the chi-square test for the independence model in the *lavaan* package. Mean interitem tetrachoric correlations ranged from .07 to .26 (median = .12), with the mean interitem correlation falling below .15 in seven of the nine datasets. Moreover, eight of the nine datasets also included at least one negative interitem correlation, with the total number of negative interitem correlations ranging from 0 to 130 (20.6%; median = 46 [7.3%]; the tetrachoric correlation matrices are available on the project’s OSF page). All correlation matrices were positive definite in the full datasets.

Item-level response rates are presented in Table S2. We compared these response rates to Baron-Cohen et al.’s (2001) criteria for item retention when developing the RMET (i.e., first criterion: at least 50% select the target response; second criterion: no more than 25% select the same incorrect response). In four of the nine datasets, at least one item failed the first criterion (range: 1–3). In all six of the datasets where we could assess the second criterion, multiple items failed this criterion (range: 2–4). Furthermore, five items failed one or both criteria across multiple datasets (Items 19 and 25 failed at least one criterion in two datasets; Item 23 in four datasets; Item 17 in five datasets; and Item 10 in six datasets).

### Single-Factor Model CFA in Full Datasets

Unsurprisingly given the large sample sizes, all single-factor models failed the chi-square test of exact fit. The results based on approximate fit indices were mixed (see Table 3). In all datasets, CFI (range: 0.46–0.86) was below the recommended level of >0.95. In contrast, apart from the Panero and Pearce datasets, the absolute fit indices indicated acceptable model fit (i.e., RMSEA and SRMR <0.08). The WLSMV fit indices, which are reported in Table 3, produced better values, as expected due to their known bias towards model acceptance; however, the general pattern of results was the same (i.e., CFI was generally unacceptable, while RMSEA was generally acceptable).

**Table 3:** Table of Fit Indices and Reliability Estimates for Single-Factor CFA Model.

Study	Type of fit index	Chi-square (χ^2^)	CFI	TLI	RMSEA (90% CI)	SRMR	Omega (ω)	Alpha (α)	Mean factor loading (range)	Items (%) with loading <0.3
Almaatouq	WLSMV	χ^2^ (594, *n* = 1,182) = 1007, *p* < .001	0.820	0.809	0.024 (0.022, 0.027)	0.070	0.70	0.70	0.364 (0.176–0.761)	8 (22%)
	ML		0.672	0.652	0.059 (0.053, 0.064)					
Dodell-Feder	WLSMV	χ^2^ (594, *n* = 9,267) = 3,600, *p* < .001	0.870	0.862	0.023 (0.023, 0.024)	0.042	0.71	0.70	0.344 (0.145–0.517)	12 (33%)
	ML		0.766	0.752	0.040 (0.039, 0.042)					
Floyd	WLSMV	χ^2^ (594, *n* = 1,016) = 915, *p* < .001	0.957	0.955	0.023 (0.020, 0.026)	0.059	0.86	0.85	0.502 (0.223–0.725)	3 (8%)
	ML		0.859	0.850	0.052 (0.046, 0.058)					
Kidd	WLSMV	χ^2^ (594, *n* = 1,112) = 808, *p* < .001	0.751	0.736	0.018 (0.015, 0.021)	0.062	0.56	0.55	0.279 (0.096–0.410)	24 (67%)
	ML		0.578	0.552	0.042 (0.036, 0.048)					
Nahal	WLSMV	χ^2^ (594, *n* = 1,168) = 926, *p* < .001	0.867	0.859	0.022 (0.019, 0.025)	0.062	0.71	0.70	0.344 (0.087–0.548)	11 (31%)
	ML		0.747	0.732	0.044 (0.039, 0.049)					
Panero	WLSMV	χ^2^ (594, *n* = 558) = 933, *p* < .001	0.900	0.894	0.032 (0.028, 0.036)	0.083	0.87	0.85	0.516 (0.208–0.743)	1 (3%)
	ML		0.644	0.623	0.101 (0.093, 0.108)					
Pearce	WLSMV	χ^2^ (594, *n* = 709) = 862, *p* < .001	0.630	0.607	0.025 (0.021, 0.029)	0.083	0.59	0.59	0.294 (0.047–0.500)	18 (50%)
	ML		0.459	0.426	0.062 (0.055, 0.069)					
Schmalor	WLSMV	χ^2^ (594, *n* = 972) = 1,002, *p* < .001	0.844	0.834	0.027 (0.024, 0.029)	0.069	0.74	0.73	0.382 (0.235–0.648)	13 (36%)
	ML		0.669	0.649	0.057 (0.052, 0.063)					
Vonk	WLSMV	χ^2^ (594, *n* = 868) = 827, *p* < .001	0.838	0.828	0.021 (0.018, 0.025)	0.066	0.67	0.66	0.329 (0.098–0.584)	17 (47%)
	ML		0.642	0.620	0.051 (0.045, 0.057)					

*Note*. CFI = comparative fit index; TLI = Tucker–Lewis index; RMSEA = root-mean-square error of approximation; SRMR = standardized root mean square residual; WLSMV = weighted least square mean and variance adjusted; ML = maximum likelihood.

The number of residual correlations with absolute values ≥.20 in each dataset ranged from 0 (Dodell-Feder dataset) to 16 (Panero dataset). Residual correlations above .20 suggest local model misfit. For example, the residual correlation between items 1 and 20 was greater than .20 across three datasets, suggesting that this pair of items may tend to covary independent of the modeled factor (see OSF page for table of all residual correlations).

Factor loadings were generally weak (see Table 3 and Table S3). The number of items within each dataset with loadings below 0.3 ranged from 1 to 24 (median = 12). Weak factor loadings indicate a weak relationship between items and the latent factor.

In combination, the weak factor loadings and low CFI values suggest that a single-factor model does not account well for RMET performance in any of the datasets, despite RMSEA and SRMR values falling within the range traditionally taken to indicate acceptable model fit.

Alpha and omega values for RMET scores based on the single-factor model are reported in Table 3. Within each dataset, the alpha and omega values were highly similar. Values indicated unacceptable levels of internal consistency in three datasets (i.e., <0.70), acceptable levels of internal consistency in four datasets (i.e., between 0.70 and 0.80), and high levels of internal consistency (i.e., >0.80) in two datasets. However, these values should be interpreted with caution given the uncertain fit of the single-factor models.

### Three-Factor Valence-Based CFA Model in Full Datasets

With the exception of the Kidd dataset, the tetrachoric correlation matrix was positive definite. Model fit indices were marginally better for a three-factor model versus a single-factor model in all datasets (see Table 4). However, model fit will often improve when additional factors are added (Stanton et al., 2023), and the overall pattern or results remained very similar to the single-factor model. In particular, the chi-square test was significant for all datasets. All approximate fit indices suggested poor model fit for the Panero dataset, while the other eight datasets had acceptable values for RMSEA and SRMR. In contrast, CFI values were below recommended levels by both the ML and WLSMV estimates for all datasets other than the Floyd dataset, where the WLSMV CFI value was 0.96. The number of residual correlations with an absolute value greater than .20, which capture the difference between each sample correlation and its value reproduced under the model, ranged from 0 to 16. The correlations between the factors were high across datasets, potentially indicating redundant factors (see Table 4).

**Table 4:** Table of Fit Indices, Factor Correlations, and Factor Loadings for Three-Factor CFA Model.

Dataset	Type of fit index	Chi-square (χ^2^)	CFI	TLI	RMSEA (90% CI)	SRMR	Positive~negative (SE)	Positive~neutral (SE)	Negative~neutral (SE)	Mean factor loading (range)	Items (%) with loading <0.3
Almaatouq	WLSMV	χ^2^ (591, *n* = 1,182) = 949, *p* < .001	0.844	0.834	0.023 (0.020, 0.025)	0.067	0.584 (0.056)	0.809 (0.048)	0.930 (0.038)	0.39 (0.18–0.92)	6 (17%)
	ML		0.724	0.706	0.054 (0.048, 0.060)						
Dodell-Feder	WLSMV	χ^2^ (591, *n* = 9,267) = 3,210, *p* < .001	0.886	0.879	0.022 (0.021, 0.023)	0.039	0.619 (0.022)	0.731 (0.019)	0.929 (0.013)	0.38 (0.17–0.56)	11 (31%)
	ML		0.797	0.784	0.038 (0.036, 0.039)						
Floyd	WLSMV	χ^2^ (591, *n* = 1,016) = 869, *p* < .001	0.963	0.960	0.022 (0.018, 0.025)	0.057	0.774 (0.042)	0.813 (0.035)	0.977 (0.016)	0.52 (0.23–0.73)	2 (6%)
	ML		0.878	0.869	0.049 (0.042, 0.055)						
Kidd	WLSMV	χ^2^ (591, *n* = 1,112) = 795, *p* < .001	0.762	0.747	0.018 (0.014, 0.021)	0.062	0.621 (0.060)	0.883 (0.084)	0.953 (0.084)	0.29 (0.16–0.45)	24 (67%)
	ML^a^		0.596^a^	0.570^a^	0.041 (0.035, 0.047)^a^						
Nahal	WLSMV	χ^2^ (591, *n* = 1,168) = 894, *p* < .001	0.878	0.870	0.021 (0.018, 0.024)	0.061	0.665 (0.060)	0.751 (0.051)	0.960 (0.036)	0.38 (0.17–0.60)	11 (31%)
	ML		0.764	0.749	0.043 (0.037, 0.048)						
Panero	WLSMV	χ^2^ (591, *n* = 558) = 883, *p* < .001	0.914	0.909	0.030 (0.026, 0.034)	0.080	0.721 (0.050)	0.861 (0.038)	0.965 (0.024)	0.54 (0.21–0.77)	1 (3%)
	ML		0.673	0.651	0.097 (0.089, 0.104)						
Pearce	WLSMV	χ^2^ (591, *n* = 709) = 849, *p* < .001	0.643	0.620	0.025 (0.021, 0.028)	0.082	0.556 (0.101)	0.819 (0.104)	0.884 (0.072)	0.32 (0.06–0.59)	16 (44%)
	ML		0.475	0.440	0.061 (0.054, 0.068)						
Schmalor	WLSMV	χ^2^ (591, *n* = 972) = 972, *p* < .001	0.854	0.844	0.026 (0.023, 0.029)	0.068	0.673 (0.054)	0.776 (0.057)	0.942 (0.032)	0.40 (0.15–0.67)	10 (28%)
	ML		0.690	0.669	0.056 (0.050, 0.061)						
Vonk	WLSMV	χ^2^ (591, *n* = 868) = 785, *p* < .001	0.865	0.856	0.019 (0.016, 0.023)	0.064	0.641 (0.077)	0.615 (0.062)	0.936 (0.050)	0.36 (0.10–0.64)	15 (42%)
	ML		0.684	0.663	0.048 (0.042, 0.054)						

*Note*. SE = standard error; CI = confidence interval; CFI = comparative fit index; TLI = Tucker–Lewis index; RMSEA = root-mean-square error of approximation; SRMR = standardized root mean square residual; WLSMV = weighted least square mean and variance adjusted; ML = maximum likelihood.

a The tetrachoric correlation matrix was not positive definite, so the ML-based estimates may be less accurate.

Similar to the single-factor models, factor loadings were generally low for the three-factor models (see Table 4 and Table S4), with the number of items with loadings below 0.3 ranging from 1 to 24 (median = 11). Thus, extracting more factors did not produce stronger loading patterns.

### EFA in Split Datasets

After dividing each dataset in half, sample sizes ranged from 279 to 4,634 participants (median: 508). The datasets used for the EFA analyses had similar mean RMET scores (range: 24.5–27.4; median: 26.6) and mean interitem correlations (range: .07–.29, median: .12) to the full samples (see Table S1). The tetrachoric correlation matrix was not positive definite for three of the split datasets (Almaatouq, Panero, and Pearce datasets). We forced *lavaan* to calculate ML-based fit indices for these models; however, these values are likely to be less accurate.

Figure S1 contains scree plots for each divided dataset. Eight of the nine scree plots are suggestive of a single-factor model, although in four of these cases, a two-factor model is also worth considering. The scree plot for the ninth dataset (Pearce dataset) most strongly suggests a two-factor model. Since we had already evaluated a single-factor model via CFA in all datasets, we conducted EFA with two factors in the five datasets where the scree plot indicated a two-factor model may be plausible (i.e., Almaatouq, Dodell-Feder, Nahal, Panero, and Pearce datasets).

A clear two-factor structure did not emerge from the five tested datasets, although the pattern of loadings across datasets was somewhat suggestive of a division between items with positive versus negative target responses (see Table 5). However, there were multiple inconsistencies. For example, item 2 (“upset”) only had a clear loading on the negative factor in two of the five datasets, while item 14 (“accusing”) loaded onto the “positive” factor in two datasets, the “negative” factor in two datasets, and neither in the fifth tested dataset. Moreover, while cross loadings were infrequent (four items had cross loadings in the Panero dataset and a single item had a cross loading in the Almaatouq and Pearce datasets), the number of items failing to load onto either factor ranged from 4 (11%) to 12 (33%), median: 10 (28%). Additionally, communalities, which indicate the proportion of each item’s variance that can be explained by all extracted factors, were generally low (see Table 5). As an exploratory analysis, we also ran the analyses using the oblimin rotation method, but this did not improve the pattern of factor loadings (see Table S5). Given the inconsistency among datasets in terms of which items loaded onto each of the two factors, the presence of multiple items in each dataset that had weak loadings on both factors, and the better theoretical fit of a single-factor model, we did not conduct CFA for two-factor models in any datasets. We report fit indices for the two-factor models in Table S6 for completeness, but these fit indices are not meaningful given that no clear two-factor structure emerged.

**Table 5:** Factor Loadings and Communalities for Two-Factor EFA Models With Default Geomin Rotation.

Dataset	Almaatouq			Dodell			Nahal			Panero			Pearce		
Item—target	Negative	Positive	*h* ^2^	Negative	Positive	*h* ^2^	Negative	Positive	*h* ^2^	Negative	Positive	*h* ^2^	Negative	Positive	*h* ^2^
R01—playful	0.00	0.27	0.07	0.01	**0.32**	0.11	−0.10	**0.37**	0.12	0.06	**0.48**	0.27	0.14	0.09	0.03
R02—upset	**0.35**	0.01	0.13	0.18	0.07	0.05	0.12	0.21	0.08	0.25	0.12	0.11	**0.53**	0.05	0.29
R03—desire	0.03	**0.40**	0.17	0.16	**0.35**	0.20	0.03	**0.42**	0.18	−0.01	**0.63**	0.39	**0.45**	0.07	0.22
R04—insisting	**0.44**	0.00	0.20	**0.41**	−0.25	0.14	**0.37**	−0.11	0.12	**0.53**	−0.01	0.27	0.06	0.13	0.02
R05—worried	**0.47**	−0.04	0.21	0.26	0.03	0.08	0.22	0.10	0.07	**0.47**	**0.37**	0.54	0.03	**0.45**	0.21
R06—fantasizing	−0.14	**0.66**	0.40	−0.03	**0.47**	0.21	−0.01	**0.55**	0.30	−0.06	**0.64**	0.38	0.23	−0.11	0.05
R07—uneasy	**0.51**	−0.18	0.23	**0.42**	−0.22	0.14	**0.50**	−0.14	0.22	**0.65**	−0.14	0.35	**−0.35**	**0.32**	0.18
R08—despondent	**0.57**	0.26	0.49	**0.34**	0.13	0.17	0.23	0.21	0.14	**0.37**	**0.38**	0.43	0.17	**0.35**	0.18
R09—preoccupied	0.12	**0.55**	0.36	**0.32**	0.25	0.23	0.27	**0.34**	0.25	**0.38**	**0.49**	0.57	0.06	**0.38**	0.16
R10—cautious	**0.33**	0.02	0.11	**0.42**	−0.21	0.15	0.27	−0.15	0.07	0.25	0.13	0.11	−0.07	**0.32**	0.10
R11—regretful	**0.47**	−0.13	0.20	**0.41**	−0.08	0.14	**0.51**	−0.16	0.23	**0.37**	**0.38**	0.43	0.17	**0.38**	0.20
R12—sceptical	**0.34**	0.09	0.14	**0.46**	−0.03	0.20	**0.39**	0.17	0.23	**0.53**	0.18	0.41	−0.09	**0.46**	0.20
R13—anticipating	0.23	0.21	0.13	**0.32**	0.12	0.15	0.24	0.21	0.14	**0.56**	0.25	0.51	−0.12	**0.33**	0.11
R14—accusing	0.21	0.24	0.14	**0.48**	0.02	0.24	0.18	**0.32**	0.17	**0.74**	0.01	0.56	**0.39**	0.21	0.23
R15—contemplative	0.28	0.29	0.22	**0.47**	0.11	0.28	**0.40**	0.21	0.26	**0.43**	0.21	0.32	−0.06	**0.50**	0.24
R16—thoughtful	**0.30**	**0.39**	0.32	0.22	0.18	0.11	0.23	0.11	0.08	0.18	**0.46**	0.33	0.06	0.20	0.05
R17—doubtful	**0.53**	−0.17	0.25	**0.36**	−0.26	0.12	**0.50**	**−0.30**	0.23	**0.66**	−0.22	0.34	−0.27	**0.32**	0.14
R18—decisive	**0.31**	0.11	0.13	**0.38**	−0.01	0.14	**0.36**	0.06	0.15	0.28	0.12	0.12	**0.33**	0.16	0.15
R19—tentative	0.23	0.04	0.06	**0.36**	0.02	0.14	**0.36**	0.23	0.24	0.29	−0.02	0.08	−0.01	0.29	0.08
R20—friendly	0.21	0.21	0.12	0.14	**0.48**	0.30	0.03	**0.36**	0.14	0.25	**0.54**	0.49	**0.37**	0.28	0.26
R21—fantasizing	0.09	**0.36**	0.16	0.05	**0.36**	0.15	0.19	**0.36**	0.22	−0.12	**0.62**	0.33	**0.46**	0.01	0.22
R22—preoccupied	**0.38**	0.24	0.27	**0.34**	0.16	0.19	0.24	0.17	0.12	**0.45**	**0.39**	0.53	0.10	**0.42**	0.20
R23—defiant	**0.41**	−0.16	0.15	**0.35**	−0.06	0.11	**0.41**	0.05	0.19	**0.67**	0.02	0.47	0.05	0.25	0.07
R24—pensive	0.26	0.29	0.21	**0.36**	0.17	0.21	**0.33**	0.20	0.20	**0.34**	**0.48**	0.52	**0.55**	−0.09	0.29
R25—interested	−0.18	**0.45**	0.18	−0.08	**0.35**	0.10	−0.27	**0.48**	0.20	−0.08	**0.60**	0.32	**0.32**	−0.09	0.10
R26—hostile	0.23	−0.06	0.05	**0.34**	0.08	0.14	**0.39**	0.27	0.30	**0.32**	0.17	0.19	0.22	0.27	0.15
R27—cautious	**0.51**	0.10	0.30	**0.36**	0.15	0.19	**0.37**	0.13	0.19	**0.57**	−0.04	0.30	−0.03	**0.42**	0.17
R28—interested	**0.35**	0.08	0.15	**0.43**	0.07	0.21	**0.49**	0.14	0.31	**0.39**	0.16	0.24	0.02	**0.52**	0.28
R29—reflective	−0.11	**0.56**	0.28	0.03	0.25	0.07	−0.02	0.18	0.03	0.06	**0.62**	0.43	0.17	0.01	0.03
R30—flirtatious	**0.38**	**0.58**	0.62	0.17	**0.42**	0.26	0.02	**0.58**	0.35	0.22	**0.69**	0.68	**0.45**	0.06	0.21
R31—confident	0.03	**0.38**	0.15	0.00	**0.32**	0.10	−0.03	0.29	0.08	−0.18	**0.60**	0.28	**0.46**	−0.05	0.21
R32—serious	0.26	0.17	0.13	0.19	**0.32**	0.19	0.08	0.22	0.07	**0.31**	**0.32**	0.29	**0.46**	−0.03	0.21
R33—concerned	0.27	0.13	0.11	0.09	0.22	0.08	0.20	0.12	0.07	0.29	**0.37**	0.33	0.09	0.15	0.04
R34—distrustful	**0.47**	0.01	0.22	**0.36**	0.07	0.16	0.29	0.14	0.13	**0.52**	0.15	0.37	−0.03	**0.46**	0.20
R35—nervous	**0.33**	−0.07	0.10	**0.41**	−0.07	0.15	**0.47**	−0.08	0.20	**0.68**	−0.19	0.37	0.04	**0.40**	0.17
R36—suspicious	0.15	0.21	0.08	**0.34**	0.05	0.13	**0.50**	0.00	0.25	**0.46**	**0.40**	0.55	0.05	0.29	0.09
Proportion variance	0.11	0.09		0.10	0.06		0.10	0.07		0.19	0.17		0.07	0.09	
Total cumulative variance	0.20			0.16			0.18			0.37			0.16		
Items with no loading >0.30	11			5			12			4			10		

*Note*. Loadings meeting the minimum threshold for an item to load onto a factor (i.e., 0.30) are presented in bold font. While no consistent two-factor structure emerged across datasets, there appeared to be a tendency for items with a negatively valenced correct response to load onto one factor and items with positively valenced correct response to load onto the other factor. *h*^2^ = communalities.

We also conducted EFAs for a three-factor model in the five datasets where the scree plots indicated the possibility of a multifactorial structure. However, no interpretable three-factor models emerged in any of the five datasets. We report the factor loadings for three-factor models in Table S7 (additionally, we report factor loadings based on oblimin rotation in Table S8). Communalities remained low for the three-factor model (see Table S9).

## Discussion

We analyzed the factor structure of RMET scores in nine large, predominantly White, non-clinical samples and found evidence of weak structural properties in all of them. We assessed two theoretically derived CFA models: a single-factor model and a three-factor valence-based model. In all nine datasets, we found evidence of poor model fit for both tested models. Moreover, we did not identify better fitting alternative factor models via EFA in any of the datasets. Given that (a) a single-factor model is the most theoretically appropriate for the RMET due to it being designed to measure a single construct and (b) scree plots were most suggestive of a single-factor model in eight of the nine datasets, we provisionally conclude that a single-factor model is the most appropriate model for RMET scores in these datasets. However, the weak factor loadings and weak interitem correlations should be considered when interpreting RMET sum scores in these datasets. Moreover, the low CFI values suggest that the single-factor model does not fit the data sufficiently better than the independence model, which is not surprising given the weak interitem correlations. Previous research has rarely examined the factor structure of RMET scores, and those few studies that have done so lacked key information required to assess model fit, found evidence of unacceptable model fit, or both (Higgins et al., 2024). Consequently, our finding that RMET scores demonstrated weak structural properties in *all* datasets that we analyzed provides important evidence that RMET scores *frequently* have weak structural properties in non-clinical samples.

The weak structural properties of RMET scores have serious implications for the interpretation of the vast and growing literature that uses RMET scores as measurements of social cognitive ability. Weak factor loadings suggest a weak relationship between the modeled factor and the test items. Moreover, mean interitem tetrachoric correlations were also weak in seven of the nine datasets. Low interitem correlations indicate that performance on one RMET item is only weakly predictive of performance on other RMET items, which is inconsistent with a single ability being primarily responsible for performance on all items. Thus, even if we were to accept a single-factor model and interpret the latent factor as social cognitive ability, the relationship between social cognitive ability and item responses would be so weak that the usefulness of the RMET for research would be open to question.

It is possible that test items are influenced by social cognitive ability, but that items also have a lot of measurement error and specific variance. In such a case, a very long test would be required to measure the construct with adequate precision for psychological research. However, at 36 items, the RMET is already a long test. Nonetheless, internal consistency estimates were below the conventionally accepted minimum value of 0.70 in three datasets and only just reached the bottom of the acceptable range in three others. This suggests that, even if items are influenced by social cognitive ability, the levels of unique variance in RMET items are so high that even a 36-item test is not long enough to make sum scores interpretable.

We can think of three issues that might contribute to the weak structural properties of RMET scores. The first issue relates to the concerns about measurement error and unique variance mentioned above. In particular, the social cognitive construct that the RMET aims to measure might be difficult to isolate using behavioral tasks (Yeung et al., 2024).^14^ In this case, the reliability of RMET items might be low due to sources of random error or unique variance. This could result in weaker interitem correlations, weaker factor loadings, and lower levels of internal consistency. Given that fit indices are differentially sensitive to weak interitem correlations and factor loadings (i.e., CFI and TLI will be biased toward indicating poor fit, while RMSEA and SRMR will be biased toward indicating good fit; Lai & Green, 2016), this could also have contributed to the mixed fit indices that we found in the datasets. Increasing the number of test items can help filter out random error and unique variance, increasing the overall reliability of a test. However, as mentioned above, at 36 items, the RMET is already a very long test. Of course, it is possible that the construct being measured is both difficult to isolate *and* multidimensional. However, we did not identify any promising multidimensional factor models in any of the datasets via EFA, and our results did not support the three-factor valence-based model that we tested, which suggests that developing an even longer version of the RMET or testing more factor models is unlikely to be productive.

A second issue that might contribute to the weak structural properties of RMET scores is that some participants might have put very little effort into the task and, thus, provided poor quality data. Indeed, most survey data likely contain at least some level of careless responding (Ward & Meade, 2023), which has been demonstrated to have significant impacts on research findings, including leading to spurious associations between variables (Stosic et al., 2024; Sulik et al., 2023; Ward & Meade, 2023; Zorowitz et al., 2023) and negatively impacting estimates of internal consistency and factor structure (Arias et al., 2020; Voss, 2024; Ward & Meade, 2023; Zorowitz et al., 2023). Notably, only three of the datasets that we analyzed (Almaatouq, Floyd, and Schmalor datasets) used attention checks to identify and exclude inattentive participants. Consequently, the datasets that did not use attention checks may have included a non-trivial proportion of participants who responded inconsistently or even randomly, which might have contributed to the weak structural properties of RMET scores in those datasets. Indeed, the fact that interitem correlations, CFI, factor loadings, and internal consistency calculated based on a single-factor model were higher for the Floyd dataset than the other datasets could be taken as tentative evidence that excluding inattentive participants may have improved data quality. However, structural properties for the Almaatouq and Schmalor datasets, which also had inattentive participants excluded, were not noticeably better than other datasets. Moreover, inspection of item-level response rates (see Table S2) reveals that there were eight easy RMET items for which the correct response was selected by over 75% of participants in each of the nine datasets. This level of consistency across the datasets would be unlikely if random responding were widespread. The mean sum scores are also very similar across datasets (range 24.5–27.5; see Table S1), which provides further evidence against substantial differences in levels of attentiveness across datasets. Overall, these patterns of results suggest that, on its own, improving the attentiveness of participants is not going to address the issue of weak structural properties of RMET scores. Nonetheless, research incorporating a variety of strategies to identify and reduce careless responding would be needed to confirm this (see Ward & Meade, 2023 for a summary of strategies for reducing careless responding).

A third issue that might contribute to the weak structural properties of RMET scores relates to potential limitations of the RMET itself as a measure of social cognition. The RMET is intended to measure some dimension/s of social cognitive ability by assessing whether people can identify complex mental states from static images of people’s eyes (i.e., “read the mind in the eyes”). However, as we have noted, the “correct” response for each item was determined via consensus among a small number of raters rather than being objectively correct; and, where item-level data has been reported for the RMET, a subset of RMET items generally fail to meet one or both of the consensus criteria that were originally used to verify the “correct” responses (Higgins et al., 2024; Higgins, Ross, Langdon, et al., 2023). Consistent with these earlier findings, in the current study we identified multiple items that failed to meet at least one of the initial consensus criteria in seven of the nine datasets. Moreover, for three of the datasets, we were only able to assess the first criterion (i.e., that greater than 50% of people selected the target response) because items were coded as correct versus incorrect rather than providing the specific response choice for each participant. As such, in these three datasets, it is possible that additional items would have failed the second criterion (i.e., more than 25% of participants might have selected the same incorrect response choice). In addition to undermining the “correctness” of the target responses for some RMET items, low levels of consensus could well reflect idiosyncratic, and thus unreliable, responding for some items.

Even in cases where the majority of participants converge on a “correct” mental state for an RMET item, evidence strongly suggests that this consensus is likely to be an artifact of the multiple-choice format of the RMET (Betz et al., 2019; Higgins et al., 2024; Higgins et al., 2025). For example, Betz et al. (2019) administered a free response version of the task where the 36 photographs from the RMET were presented one at a time without any mental state descriptors and participants were asked to generate their own responses. Notably, less than 10% of participant-generated responses were similar in meaning to the “correct” response in the multiple-choice format of the test, and only approximately 40% of participant-generated responses even had the same valence as the target response. These results strongly suggest that people do not identify the “correct” complex mental states in the RMET directly from the photographs of people’s eyes. Rather, as Betz et al. (2019) argue, the words provide additional context that guides responding.

Building on this critique, Higgins et al. (2025) argue that for some RMET items, the cues in the photographs are insufficient to isolate the target response as the “correct” response and that RMET performance likely relies on abilities other than emotion recognition, such as vocabulary, abductive reasoning, and problem-solving skills (e.g., participants might use process of elimination to rule out implausible options before guessing between any remaining plausible options). If RMET performance relies on a variety of abilities, it would impact both the factor structure and internal consistency of RMET scores. Interestingly, Higgins et al. (2025) also observe that there was a high level of variability in the participant-generated responses collected by Betz et al. (2019) and argue that many “incorrect” participant-generated responses were at least as plausible as the “correct” responses in the multiple-choice RMET. Thus, it is possible that people who respond “incorrectly” to an RMET item may, nonetheless, be able to generate plausible mental states based on the photographs for that item, in which case RMET scores are unlikely to measure the ability to recognize mental states.

### Limitations

Our study has several limitations. First, as previously mentioned, coding of the participants’ specific response choices was not available for three of the nine datasets. As such, we were not able to assess the second inclusion criterion from the original validation study in these datasets. Second, the datasets we reanalyzed were drawn from convenience samples and comprise participants who were predominantly White (when ethnicity was specified) and from the United States, the United Kingdom, and Canada, which raises concerns about the generalizability of our results to other populations (Henrich et al., 2010). The lack of racial and ethnic diversity in our samples is particularly relevant given that all 36 items in the RMET show White eyes, which could well contribute to differences in the structural properties of RMET scores when used with participants from different ethnic and racial groups (e.g., Van Staden & Callaghan, 2022). Third, while the RMET is frequently used in clinical research (Higgins et al., 2024), our study did not include any data from clinical samples. Determining whether RMET scores demonstrate acceptable structural properties in clinical groups is vital for assessing the credibility of existing clinical research based on RMET scores. Critically, if the weak structural properties that we found in the current study are the result of limitations of the RMET itself as a measure of social cognitive ability, then RMET scores may exhibit unacceptably weak structural properties in other populations too, including clinical populations.

## Conclusion

We examined the structural properties of RMET scores in nine large, non-clinical samples. We found that factor analysis revealed inadequate support for all tested unidimensional and multidimensional models; interitem correlations were weak; internal consistency was difficult to assess given the unclear factor structure; and five items did not consistently meet the original consensus criteria across datasets. Overall, these findings raise serious doubts about the adequacy of the structural properties of RMET sum scores and, thus, lend additional support to the argument that RMET scores are unsubstantiated as measurements of social cognitive ability (Higgins et al., 2024, 2025).

More broadly, our findings underscore the importance of routinely reporting sample-specific evidence for the structural properties of test scores (Appelbaum et al., 2018), which provides a key source of information for assessing the credibility of research findings. Our study also provides an example of how structural properties can be examined retrospectively by re-analyzing raw data from existing datasets. We hope that others will follow our lead in assessing the adequacy of the structural properties of scores from other widely used psychological tests for which there is currently limited structural validity evidence.

## Supplemental Material

sj-docx-1-asm-10.1177_10731911251328604 – Supplemental material for Reading the Mind in the Eyes Test Scores Demonstrate Poor Structural Properties in Nine Large Non-Clinical Samples Supplemental material, sj-docx-1-asm-10.1177_10731911251328604 for Reading the Mind in the Eyes Test Scores Demonstrate Poor Structural Properties in Nine Large Non-Clinical Samples by Wendy C. Higgins, Victoria Savalei, Vince Polito and Robert M. Ross in Assessment

## Notes

1. In relation to this point, see Higgins, Savalei, et al.’s (2023) commentary on Greenberg et al. (2023), which argues that, despite a huge sample of more than 300,000 participants, the omega reliability estimates reported by Greenberg and colleagues are uninterpretable because no information is provided about the factor model that was used to calculate the omega estimates.

2. Some studies reported internal consistency metrics for more than one sample and/or reported more than one type of estimate for a sample. This is why the number of estimates and numbers of studies reporting estimates are different.

3. The role of reliability is different when scores are used to predict outcomes rather than to measure specific constructs. In the former case, low levels of internal consistency can be acceptable (Revelle, 2024).

4. As noted in our pre-registration, we did not request data from Warrier et al. (2018) or Warrier and Baron-Cohen (2018), despite matching our inclusion criteria. This was because the data were collected via 23andMe, which only provides summary level data.

5. The data from Floyd and Woo (2020) and Vonk et al. (2015) are not available on the project’s OSF page because we were not granted permission to share them. Instead, these datasets can be requested directly from the corresponding authors of those studies.

6. Panero et al. (2016) analyzed data collected by three different research groups. We were only able to obtain the data from one of these research groups. Moreover, the data from this research group contained RMET scores collected from four separate studies. We have combined the data from these four studies into a single dataset, matching what was done in the original study (although the combined dataset in the original study also included the RMET scores collected by the other two research groups).

7. These alpha values are all within .01 of the values we calculate in the current study (see Table 3).

8. In our pre-registration, we stated that we would assess the degree of nonnormality in the distribution of RMET sum scores by computing skew and kurtosis. However, this was a mistake because items are binary, not continuous. Consequently, we do not report this analysis.

9. When we pre-registered our study on July 2, 2022, the *efa* function in the *lavaan* package had not been released (it was released in version 6-13 on January 9, 2023). Despite having pre-registered that we would conduct EFA using the *psych* package, we deviated from our pre-registration and used the *efa* function in the *lavaan* package. This function can conduct EFA with categorical data using WLSMV estimation, which allowed for consistency in estimator choice between our CFA and EFA analyses.

10. We pre-registered the use of the *n_factors* function in the *parameters* package (Lüdeck et al., 2020) to determine the number of factors to retain. However, this was a mistake because the *n-factors* function does not allow for the specification of data as categorical. As an alternative, we relied on scree plots. We also used parallel analysis, which has been recommended as one of the most accurate methods for determining how many factors to retain (e.g., Auerswald & Moshagen, 2019). However, parallel analysis can be less accurate when factor loadings are low (Auerswald & Moshagen, 2019; Lim & Jahng, 2019) or when data are binary rather than continuous (Tran & Formann, 2009). Nonetheless, in all nine datasets, parallel analysis and the screen plots led to very similar conclusions about the number of factors to extract; see Figure S2.

11. Following the advice of Kline (2023), we did not calculate the normed chi-square for factor models, despite pre-registering that we would report it, because this statistic has “little statistical or logical foundation” (p. 161).

12. We report both CFI and TLI in the tables of fit indices for each model. However, we only discuss CFI because, as noted by Kline (2023), the two indices produce very similar results, which makes it redundant to discuss both.

13. The *compRelSEM* function replaces the now depreciated *reliability* function, which we pre-registered an intention to use. In our scripts on the project’s OSF page, we provide code for calculating these versions of alpha and omega using the depreciated *reliability* function in the *semTools* package. In all datasets, both functions returned the same values for alpha and omega.

14. Social cognitive ability is generally considered to be multifaceted (Quesque et al., 2024; Schaafsma et al., 2015), and the RMET, which focuses narrowly on the ability to recognize mental states from static, decontextualized photographs, was not designed to capture all dimensions of social cognitive ability (Baron-Cohen et al., 2001). However, here, we are suggesting that the specific dimension of social cognitive ability that the RMET is intended to measure may be difficult to isolate using behavioral tasks.

## References

[bibr1-10731911251328604] *Almaatouq A. Alsobay M. Yin M. Watts D. J. (2021). Task complexity moderates group synergy. Proceedings of the National Academy of Sciences of the United States of America, 118(36), e2101062118. 10.1073/pnas.2101062118 PMC8433503 34479999

[bibr2-10731911251328604] Appelbaum M. Cooper H. Kline R. B. Mayo-Wilson E. Nezu A. M. Rao S. M. (2018). Journal article reporting standards for quantitative research in psychology: The APA Publications and Communications Board task force report. American Psychologist, 73(1), 3. 10.1037/amp0000191 29345484

[bibr3-10731911251328604] Arias V. B. Garrido L. E. Jenaro C. Martínez-Molina A. Arias B. (2020). A little garbage in, lots of garbage out: Assessing the impact of careless responding in personality survey data. Behavior Research Methods, 52(6), 2489–2505. 10.3758/s13428-020-01401-8 32462604

[bibr4-10731911251328604] Auerswald M. Moshagen M. (2019). How to determine the number of factors to retain in exploratory factor analysis: A comparison of extraction methods under realistic conditions. Psychological Methods, 24(4), 468–491. 10.1037/met0000200 30667242

[bibr5-10731911251328604] Bandalos D. L. Finney S. J. (2018). Factor analysis: Exploratory and confirmatory. In Hancock G. R. Stapleton L. M. Mueller R. O. (Eds.), The reviewer’s guide to quantitative methods in the social sciences (pp. 98–122). Routledge.

[bibr6-10731911251328604] Baron-Cohen S. Wheelwright S. Hill J. Raste Y. Plumb I. (2001). The “Reading the Mind in the Eyes” Test revised version: A study with normal adults, and adults with Asperger syndrome or high-functioning autism. Journal of Child Psychology and Psychiatry, 42(2), 241–251. 10.1017/S0021963001006643 11280420

[bibr7-10731911251328604] Bartlett M. S. (1954). A note on the multiplying factors for various χ^2^ approximations. Journal of the Royal Statistical Society. Series B, Methodological, 16(2), 296–298. 10.1111/j.2517-6161.1954.tb00174.x

[bibr8-10731911251328604] Bergh D. (2015). Sample size and chi-squared test of fit—A comparison between a random sample approach and a chi-square value adjustment method using Swedish adolescent data. In Q. Zhang & H. Yang (Eds.), Pacific Rim Objective Measurement Symposium (PROMS) 2014 Conference Proceedings (pp. 197–211). Springer Berlin/Heidelberg. 10.1007/978-3-662-47490-7_15

[bibr9-10731911251328604] Betz N. Hoemann K. Barrett L. F. (2019). Words are a context for mental inference. Emotion, 19(8), 1463–1477. 10.1037/emo0000510 30628815 PMC6620159

[bibr10-10731911251328604] *Carroll G. A. Montrose V. T. Burke T. (2021). Correlates of social cognition and psychopathic traits in a community-based sample of males. Frontiers in Psychology, 12, 656299. 10.3389/fpsyg.2021.656299 33995215 PMC8120153

[bibr11-10731911251328604] Clark L. A. Watson D. (2019). Constructing validity: New developments in creating objective measuring instruments. Psychological Assessment, 31(12), 1412–1427. 10.1037/pas0000626 30896212 PMC6754793

[bibr12-10731911251328604] *De Paoli T. Fuller-Tyszkiewicz M. Huang C. Krug I. (2020). A network analysis of borderline personality disorder symptoms and disordered eating. Journal of Clinical Psychology, 76(4), 787–800. 10.1002/jclp.22916 31953849

[bibr13-10731911251328604] *Dodell-Feder D. Ressler K. J. Germine L. T. (2020). Social cognition or social class and culture? On the interpretation of differences in social cognitive performance. Psychological Medicine, 50(1), 133. 10.1017/S003329171800404X 30616706

[bibr14-10731911251328604] Flake J. K. Fried E. I. (2020). Measurement schmeasurement: Questionable measurement practices and how to avoid them. Advances in Methods and Practices in Psychological Science, 3(4), 456–465. https://doi.org/https://doi.org/10.1177/2515245920952393

[bibr15-10731911251328604] Flake J. K. Pek J. Hehman E. (2017). Construct validation in social and personality research: Current practice and recommendations. Social Psychological and Personality Science, 8(4), 370–378. 10.1177/1948550617693063

[bibr16-10731911251328604] Flora D. B. (2020). Your coefficient alpha is probably wrong, but which coefficient omega Is right? A tutorial on using R to obtain better reliability estimates. Advances in Methods and Practices in Psychological Science, 3(4), 484–501. 10.1177/2515245920951747

[bibr17-10731911251328604] *Floyd K. Woo N. T. (2020). Loneliness and social monitoring: A conceptual replication of Knowles et al. Personal Relationships, 27(1), 209–223. 10.1111/pere.12304

[bibr18-10731911251328604] *Germine L. Dunn E. C. McLaughlin K. A. Smoller J. W. (2015). Childhood adversity is associated with adult theory of mind and social affiliation, but not face processing. PLoS One, 10(6), e0129612. 10.1371/journal.pone.0129612 PMC4466913 26068107

[bibr19-10731911251328604] Green S. B. Yang Y. (2009). Reliability of summed item scores using structural equation modeling: An alternative to coefficient alpha. Psychometrika, 74(1), 155–167. 10.1007/s11336-008-9099-3

[bibr20-10731911251328604] Greenberg D. M. Warrier V. Abu-Akel A. Allison C. Gajos K. Z. Reinecke K. Rentfrow P. J. Radecki M. A. Baron-Cohen S. (2023). Sex and age differences in “theory of mind” across 57 countries using the English version of the “Reading the Mind in the Eyes” Test. Proceedings of the National Academy of Sciences, 120(1), e2022385119. 10.1073/pnas.2022385119 PMC9910622 36584298

[bibr21-10731911251328604] Hair J. F. (2019). Multivariate data analysis (8th ed.). Cengage.

[bibr22-10731911251328604] Hair J. F. Anderson R. E. Babin B. J. Black W. C. (2010). Multivariate data analysis: A global perspective (7th ed.). Pearson.

[bibr23-10731911251328604] Harkness K. L. Sabbagh M. A. Jacobson J. A. Chowdrey N. K. Chen T. (2005). Enhanced accuracy of mental state decoding in dysphoric college students. Cognition and Emotion, 19(7), 999–1025. 10.1080/02699930541000110

[bibr24-10731911251328604] *Hartshorne J. K. Germine L. T. (2015). When does cognitive functioning peak? The asynchronous rise and fall of different cognitive abilities across the life span. Psychological Science, 26(4), 433–443. 10.1177/0956797614567339 25770099 PMC4441622

[bibr25-10731911251328604] Henrich J. Heine S. J. Norenzayan A. (2010). The weirdest people in the world? Behavioral and Brain Sciences, 33(2–3), 61–83. 10.1017/S0140525X0999152X 20550733

[bibr26-10731911251328604] Higgins W. C. Kaplan D. M. Deschrijver E. Ross R. M. (2024). Construct validity evidence reporting practices for the Reading the Mind in the Eyes Test: A systematic scoping review. Clinical Psychology Review, 108, 102378. https://doi.org/https://doi.org/10.1016/j.cpr.2023.102378 38232573 10.1016/j.cpr.2023.102378

[bibr27-10731911251328604] Higgins W. C. Kaplan D. M. Deschrijver E. Ross R. M. (2025). Why most research based on the Reading the Mind in the Eyes Test is unsubstantiated and uninterpretable: A response to Murphy and Hall (2024). Clinical Psychology Review, 115, 102530. 10.1016/j.cpr.2024.102530 39701014

[bibr28-10731911251328604] Higgins W. C. Ross R. M. Langdon R. Polito V. (2023). The “Reading the Mind in the Eyes” Test shows poor psychometric properties in a large, demographically representative U.S. sample. Assessment, 30(6), 1777–1789. 10.1177/10731911221124342 36124391

[bibr29-10731911251328604] Higgins W. C. Ross R. M. Polito V. Kaplan D. M. (2023). Three threats to the validity of the Reading the Mind in the Eyes test: A commentary on Pavolova and Sokolov (2022). Neuroscience and Biobehavioral Reviews, 147, 105088. 10.1016/j.neubiorev.2023.105088 36787872

[bibr30-10731911251328604] Higgins W. C. Savalei V. Polito V. Ross R. M. (2023). Validation of the reading the mind in the eyes test requires an interpretable factor model. Proceedings of the National Academy of Sciences of the United States of America, 120(52), e2303706120. 10.1073/pnas.2303706120 PMC10756292 38109524

[bibr31-10731911251328604] Hudson C. C. Shamblaw A. L. Harkness K. L. Sabbagh M. A. (2020). Valence in the reading the mind in the eyes task. Psychological Assessment, 32(7), 623–634. 10.1037/pas0000818 32237882

[bibr32-10731911251328604] Johnston L. Miles L. McKinlay A. (2008). A critical review of the Eyes Test as a measure of social-cognitive impairment. Australian Journal of Psychology, 60(3), 135–141. 10.1080/00049530701449521

[bibr33-10731911251328604] Jorgensen T. D. Pornprasertmanit S. Schoemann A. M. Rosseel Y. (2022). semTools: Useful tools for structural equation modeling. R package version .5-6. https://CRAN.R-project.org/package=se

[bibr34-10731911251328604] Kaiser H. F. (1974). An index of factorial simplicity. Psychometrika, 39(1), 31–36. 10.1007/BF02291575

[bibr35-10731911251328604] *Kidd D. Castano E. (2017). Different stories: How levels of familiarity with literary and genre fiction relate to mentalizing. Psychology of Aesthetics, Creativity, and the Arts, 11(4), 474–486. 10.1037/aca0000069

[bibr36-10731911251328604] Kim H. A. Kaduthodil J. Strong R. W. Germine L. T. Cohan S. Wilmer J. B. (2024). Multiracial Reading the Mind in the Eyes Test (MRMET): An inclusive version of an influential measure. Behavior Research Methods, 56(6), 5900–5917. 10.3758/s13428-023-02323-x 38630159 PMC11335804

[bibr37-10731911251328604] Kline R. B. (2023). Principles and practice of structural equation modeling (5th ed.). Guilford.

[bibr38-10731911251328604] Lai K. Green S. B. (2016). The problem with having two watches: Assessment of fit when RMSEA and CFI disagree. Multivariate Behavioral Research, 51(2–3), 220–239. 10.1080/00273171.2015.1134306 27014948

[bibr39-10731911251328604] Lim S. Jahng S. (2019). Determining the number of factors using parallel analysis and its recent variants. Psychological Methods, 24(4), 452–467. 10.1037/met0000230 31180694

[bibr40-10731911251328604] Lüdecke D. Ben-Shachar M. Patil I. Makowski D. (2020). Extracting, computing and exploring the parameters of statistical models using R. Journal of Open Source Software, 5(53), 2445. https://doi.org/1.21105/joss.02445

[bibr41-10731911251328604] Marsh H. W. Hau K.-T. Wen Z. (2004). In search of golden rules: Comment on hypothesis-testing approaches to setting cutoff values for fit indexes and dangers in overgeneralizing Hu and Bentler’s (1999) findings. Structural Equation Modeling, 11(3), 320–341. 10.1207/s15328007sem1103_2

[bibr42-10731911251328604] McNeish D. (2024). Practical implications of sum scores being psychometrics’ greatest accomplishment. Psychometrika, 89(4), 1148–1169. 10.1007/s11336-024-09988-z 39031300

[bibr43-10731911251328604] McNeish D. Wolf M. G. (2023). Dynamic fit index cutoffs for confirmatory factor analysis models. Psychological Methods, 28(1), 61–88. 10.1037/met0000425 34694832

[bibr44-10731911251328604] Miles J. Shevlin M. (2007). A time and a place for incremental fit indices. Personality and Individual Differences, 42(5), 869–874. 10.1016/j.paid.2006.09.022

[bibr45-10731911251328604] Murphy B. A. Hall J. A. (2024). How a strong measurement validity review can go astray: A look at Higgins et al. (2024) and recommendations for future measurement-focused reviews. Clinical Psychology Review, 114, 102506. 10.1016/j.cpr.2024.102506 39615954

[bibr46-10731911251328604] *Nahal P. Hurd P. L. Read S. Crespi B. (2021). Cognitive empathy as imagination: Evidence from reading the mind in the eyes in autism and schizotypy. Frontiers in psychiatry, 12, 665721. 10.3389/fpsyt.2021.665721 33868063 PMC8047060

[bibr47-10731911251328604] O’Connor B. P. (2023). EFA.dimensions: Exploratory factor analysis functions for assessing dimensionality. R package version 1.7.9. https://CRAN.R-project.org/package=EFA.dimensions

[bibr48-10731911251328604] Oakley B. F. Brewer R. Bird G. Catmur C. (2016). Theory of mind is not theory of emotion: A cautionary note on the reading the Mind in the Eyes Test . Journal of Abnormal Psychology, 125(6), 818–823. 10.1037/abn0000182 27505409 PMC4976760

[bibr49-10731911251328604] Olderbak S. Wilhelm O. Olaru G. Geiger M. Brenneman M. W. Roberts R. D. (2015). A psychometric analysis of the Reading the Mind in the Eyes Test: Toward a brief form for research and applied settings. Frontiers in Psychology, 6, 1503. 10.3389/fpsyg.2015.01503 26500578 PMC4593947

[bibr50-10731911251328604] *Panero M. E. Weisberg D. S. Black J. Goldstein T. R. Barnes J. L. Brownell H. Winner E. (2016). Does reading a single passage of literary fiction really improve theory of mind? An attempt at replication. Journal of Personality and Social Psychology, 111(5), e46–e54. 10.1037/pspa0000064 27642659

[bibr51-10731911251328604] Patil I. Makowski D. Ben-Shachar M. Wiernik B. Bacher E. Lüdecke D. (2022). datawizard: An R package for easy data preparation and statistical transformations. Journal of Open Source Software, 7(78), 4684. 10.21105/joss.04684.

[bibr52-10731911251328604] *Pearce E. Wlodarski R. Machin A. Dunbar R. I. M. (2019a). Exploring the links between dispositions, romantic relationships, support networks and community inclusion in men and women. PLoS One, 14(5), e0216210. 10.1371/journal.pone.0216210 PMC6504087 31063463

[bibr53-10731911251328604] *Pearce E. Wlodarski R. Machin A. Dunbar R. I. M. (2019b). Genetic influences on social relationships: Sex differences in the mediating role of personality and social cognition. Adaptive Human Behavior and Physiology, 5(4), 331–351. 10.1007/s40750-019-00120-5

[bibr54-10731911251328604] Posit Team. (2023). RStudio: Integrated development environment for R. Posit Software, PBC. http://www.posit.co/

[bibr55-10731911251328604] Quesque F. Apperly I. Baillargeon R. Baron-Cohen S. Becchio C. Bekkering H. Bernstein D. Bertoux M. Bird G. Bukowski H. Burgmer P. Carruthers P. Catmur C. Dziobek I. Epley N. Erle T. M. Frith C. Frith U. Galang C. M. … Brass M. (2024). Defining key concepts for mental state attribution. Communications Psychology, 2(1), 29. 10.1038/s44271-024-00077-6 39242813 PMC11332223

[bibr56-10731911251328604] R Core Team. (2022). R: A language and environment for statistical computing. R Foundation for Statistical Computing. https://www.R-project.org/

[bibr57-10731911251328604] Revelle W. (2023). psych: Procedures for psychological, psychometric, and personality research. Northwestern University. R package version 2.3.9, https://CRAN.R-project.org/package=psych

[bibr58-10731911251328604] Revelle W. (2024). The seductive beauty of latent variable models: Or why I don’t believe in the Easter Bunny. Personality and Individual Differences, 221, 112552. 10.1016/j.paid.2024.112552

[bibr59-10731911251328604] Rosseel Y. (2012). lavaan: An R package for structural equation modeling. Journal of Statistical Software, 48(2), 1–36. https://doi.org/1.18637/jss.v048.i02

[bibr60-10731911251328604] Saris W. E. Satorra A. van der Veld W. M. (2009). Testing structural equation models or detection of misspecifications? Structural Equation Modeling, 16(4), 561–582. 10.1080/10705510903203433

[bibr61-10731911251328604] Savalei V. (2012). The relationship between root mean square error of approximation and model misspecification in confirmatory factor analysis models. Educational and Psychological Measurement, 72(6), 910–932. 10.1177/0013164412452564

[bibr62-10731911251328604] Savalei V. (2021). Improving fit indices in structural equation modeling with categorical data. Multivariate Behavioral Research, 56(3), 390–407. 10.1080/00273171.2020.1717922 32054327

[bibr63-10731911251328604] Savalei V. Reise S. P. (2019). Don’t forget the model in your model-based reliability coefficients: A reply to McNeish (2018). Collabra: Psychology, 5(1), 36. 10.1525/collabra.247

[bibr64-10731911251328604] Schaafsma S. M. Pfaff D. W. Spunt R. P. Adolphs R. (2015). Deconstructing and reconstructing theory of mind. Trends in Cognitive Sciences, 19(2), 65–72. 10.1016/j.tics.2014.11.007 25496670 PMC4314437

[bibr65-10731911251328604] *Schmalor A. Heine S. J. (2021). Subjective economic inequality decreases emotional intelligence, especially for people of high social class. Social Psychological and Personality Science, 13(2), 608–617. 10.1177/19485506211024024 35251492 PMC8892066

[bibr66-10731911251328604] Silverman C. (2022). How to read “Reading the Mind in the Eyes.” Notes and records of the Royal Society of London, 76(4), 683–697. 10.1098/rsnr.2021.0058

[bibr67-10731911251328604] Slaney K. (2017). Validating psychological constructs historical, philosophical, and practical dimensions. Palgrave Macmillan.

[bibr68-10731911251328604] Slaney K. L. Maraun M. D. (2008). A proposed framework for conducting data-based test analysis. Psychological Methods, 13(4), 376–390. 10.1037/a0014269 19072000

[bibr69-10731911251328604] Stanton K. Watts A. L. Levin-Aspenson H. F. Carpenter R. W. Emery N. N. Zimmerman M. (2023). Focusing narrowly on model fit in factor analysis can mask construct heterogeneity and model misspecification: Applied demonstrations across sample and assessment types. Journal of Personality Assessment, 105(1), 1–13. 10.1080/00223891.2022.2047060 35286224

[bibr70-10731911251328604] Steiger J. H. (2000). Point estimation, hypothesis testing, and interval estimation using the RMSEA: Some comments and a reply to Hayduk and Glaser. Structural Equation Modeling, 7(2), 149–162. 10.1207/S15328007SEM0702_1

[bibr71-10731911251328604] Stosic M. D. Murphy B. A. Duong F. Fultz A. A. Harvey S. E. Bernieri F. (2024). Careless responding: Why many findings are spurious or spuriously inflated. Advances in Methods and Practices in Psychological Science, 7(1), 25152459241231581. 10.1177/25152459241231581

[bibr72-10731911251328604] Sulik J. Ross R. M. Balzan R. McKay R. (2023). Delusion-like beliefs and data quality: Are classic cognitive biases artifacts of carelessness? Journal of Psychopathology and Clinical Science, 132(6), 749–760. 10.1037/abn0000844 37326560

[bibr73-10731911251328604] Tran U. S. Formann A. K. (2009). Performance of parallel analysis in retrieving unidimensionality in the presence of binary data. Educational and Psychological Measurement, 69(1), 50–61. 10.1177/0013164408318761

[bibr74-10731911251328604] Van Staden J. G. Callaghan C. W. (2022). An evaluation of the reading the mind in the eyes test’s psychometric properties and scores in South Africa—cultural implications. Psychological Research, 86(7), 2289–2300. 10.1007/s00426-021-01539-w 34125281

[bibr75-10731911251328604] *Vonk J. Zeigler-Hill V. Ewing D. Mercer S. Noser A. E. (2015). Mindreading in the dark: Dark personality features and theory of mind. Personality and Individual Differences, 87, 50–54. 10.1016/j.paid.2015.07.025

[bibr76-10731911251328604] Voss N. M. (2024). The effects of careless responding on the fit of confirmatory factor analysis and item response theory models. Behavior Research Methods, 56(2), 577–599. 10.3758/s13428-023-02074-9 36737580

[bibr77-10731911251328604] Ward M. K. Meade A. W. (2023). Dealing with careless responding in survey data: Prevention, identification, and recommended best practices. Annual Review of Psychology, 74(1), 577–596. 10.1146/annurev-psych-040422-045007 35973734

[bibr78-10731911251328604] Warrier V. Baron-Cohen S. (2018). Genetic contribution to “theory of mind” in adolescence. Scientific Reports, 8(1), 3465–3469. 10.1038/s41598-018-21737-8 29472613 PMC5823893

[bibr79-10731911251328604] Warrier V. Grasby K. L. Uzefovsky F. Toro R. Smith P. Chakrabarti B. Khadake J. Mawbey-Adamson E. Litterman N. Hottenga J. J. Lubke G. Boomsma D. I. Martin N. G. Hatemi P. K. Medland S. E. Hinds D. A. Bourgeron T. Baron-Cohen S. (2018). Genome-wide meta-analysis of cognitive empathy: Heritability, and correlates with sex, neuropsychiatric conditions and cognition. Molecular Psychiatry, 23(6), 1402–1409. 10.1038/mp.2017.122 28584286 PMC5656177

[bibr80-10731911251328604] *Weisman O. Pelphrey K. A. Leckman J. F. Feldman R. Lu Y. Chong A. Chen Y. Monakhov M. Chew S. H. Ebstein R. P. (2015). The association between 2D:4D ratio and cognitive empathy is contingent on a common polymorphism in the oxytocin receptor gene (OXTR rs53576). Psychoneuroendocrinology, 58, 23–32. 10.1016/j.psyneuen.2015.04.007 25935637

[bibr81-10731911251328604] Widaman K. F. Revelle W. (2023). Thinking thrice about sum scores, and then some more about measurement and analysis. Behavior Research Methods, 55(2), 788–806. 10.3758/s13428-022-01849-w 35469086 PMC10027776

[bibr82-10731911251328604] Yeung E. K. L. Apperly I. A. Devine R. T. (2024). Measures of individual differences in adult theory of mind: A systematic review. Neuroscience and Biobehavioral Reviews, 157, 105481. 10.1016/j.neubiorev.2023.105481 38036161

[bibr83-10731911251328604] Zhang L. Song Y. Liu L. Liu J. (2016). Dissociable roles of internal feelings and face recognition ability in facial expression decoding. NeuroImage, 132, 283–292. 10.1016/j.neuroimage.2016.02.049 26908317

[bibr84-10731911251328604] Zorowitz S. Solis J. Niv Y. Bennett D. (2023). Inattentive responding can induce spurious associations between task behaviour and symptom measures. Nature Human Behaviour, 7(10), 1667–1681. 10.1038/s41562-023-01640-7 PMC11170515 37414886

