# Supplementary material for: Reading the Mind in the Eyes Test Scores Demonstrate Poor Structural Properties in Nine Large Non-Clinical Samples
Source: Assessment. 2025 Mar 29;33(2):204–20. doi: 10.1177/10731911251328604 (PMC12824027; doi:10.1177/10731911251328604)
Supplement: sj-docx-1-asm-10.1177_10731911251328604 – Supplemental material for Reading the Mind in the Eyes Test Scores Demonstrate Poor Structural Properties in Nine Large Non-Clinical Samples [file sj-docx-1-asm-10.1177_10731911251328604.docx]

## Supplementary Materials

**Table S1**

*Descriptive Statistics for the RMET*

| Dataset | Mean RMET (SD) | Skew | Kurtosis | AIC -Tetrachoric (range) | Number (%) of correlations above .15 | KMO - MSA | MSA interpretation^i^ | BTS | Test of the independence model | Mean RMET (SD) in split dataset used for EFA | AIC – Tetrachoric (range) in split dataset used for EFA |
| --- | --- | --- | --- | --- | --- | --- | --- | --- | --- | --- | --- |
| Almaatouq | 27.7 (4.3) | -1.1 | 5.2 | .13 (-.11, .51) | 255 (40%) | .69 | mediocre | Pass | Pass | 27.4 (4.5) | .13 (-.16, .49) |
| Dodell-Feder | 27.5 (4.7) | -.9 | 4.3 | .12 (-.13, .30) | 193 (31%) | .90 | marvellous | Pass | Pass | 26.6 (4.5) | .12 (-.14, .29) |
| Floyd | 26.4 (6.1) | -1.2 | 4.0 | .24 (-.06, .55) | 493 (78%) | .90 | marvellous | Pass | Pass | 26.0 (6.3) | .26 (-.07, .56) |
| Kidd | 26.5 (3.8) | -.3 | 3.0 | .07 (-.21, .26) | 65 (10%) | .57 | miserable | Pass | Pass | 26.6 (3.8) | .07 (-.30, .35) |
| Nahal | 26.5 (4.6) | -1.0 | 4.1 | .12 (-.15, .37) | 228 (36%) | .77 | middling | Pass | Pass | 26.6 (4.6) | .12 (-.22, 37) |
| Panero | 26.4 (6.2) | -1.4 | 5.1 | .26 (-.19, .61) | 506 (80%) | .61 | mediocre | Pass | Pass | 25.8 (6.7) | .29 (-.15, .69) |
| Pearce | 27.5 (3.8) | -.8 | 4.3 | .08 (-.21, .36) | 135 (21%) | .55 | miserable | Pass | Pass | 27.3 (4.0) | .08 (-.29, .39) |
| Schmalor | 25.6 (4.9) | -.98 | 4.3 | .13 (-.21, .48) | 270 (43%) | .76 | middling | Pass | Pass | 25.7 (4.7) | .11 (-.26, .44) |
| Vonk | 24.5 (4.5) | -.8 | 4.1 | .10 (-.16, 35) | 158 (25%) | .64 | mediocre | Pass | Pass | 24.5 (4.1) | .07 (-.28, .43) |

*Note.* RMET = Reading the Mind in the Eyes Test. AIC = average interitem correlation based on tetrachoric correlation matrix KMO - MSA = Kaiser-Meyer-Olkin – Measure of sampling adequacy. BTS = Bartlett’s test of sphericity. EFA = exploratory factor analysis. ^i^ The terms describing the interpretations of the MSA originally come from Kaiser, H. F. (1974) An index of factorial simplicity. *Psychometrika*, *39*(1), 31-36. <https://doi.org/10.1007/BF02291575>.

**Table S2**

*Item Level Response Rates*

| **RMET item** | **Almaatouq** | **Dodell-Feder** | | **Floyd** | **Kidd** | **Nahal** | **Panero** | **Pearce** | **Schmalor** | **Vonk** |
| --- | --- | --- | --- | --- | --- | --- | --- | --- | --- | --- |
| 1 |  |  | |  |  |  |  |  |  |  |
| **playful** | .74 | .67 | | .83 | .88 | .63 | .76 | .73 | .53 | .52 |
| comforting | .11 | .08 | | .06 | .04 |  | .07 |  | .09 |  |
| irritated | .09 | .23 | | .09 | .06 |  | .14 |  | .**31** |  |
| bored | .06 | .02 | | .01 | .02 |  | .02 |  | .06 |  |
| 2 |  |  | |  |  |  |  |  |  |  |
| **upset** | .73 | .77 | | .70 | .72 | .75 | .70 | .77 | .70 | .74 |
| terrified | .14 | .07 | | .17 | .23 |  | .14 |  | .13 |  |
| arrogant | .02 | .04 | | .02 | .00 |  | .01 |  | .02 |  |
| annoyed | .11 | .13 | | .10 | .04 |  | .15 |  | .15 |  |
| 3 |  |  | |  |  |  |  |  |  |  |
| **desire** | .85 | .87 | | .69 | .64 | .84 | .88 | .83 | .84 | .79 |
| joking | .03 | .02 | | .05 | .07 |  | .02 |  | .03 |  |
| flustered | .04 | .04 | | .10 | .11 |  | .04 |  | .06 |  |
| convinced | .08 | .07 | | .16 | .18 |  | .06 |  | .08 |  |
| 4 |  |  | |  |  |  |  |  |  |  |
| **insisting** | .82 | .73 | | .80 | .75 | .74 | .72 | .76 | .78 | .62 |
| joking | .01 | .01 | | .02 | .05 |  | .03 |  | .02 |  |
| amused | .07 | .10 | | .10 | .10 |  | .10 |  | .09 |  |
| relaxed | .10 | .16 | | .07 | .10 |  | .15 |  | .12 |  |
| 5 |  |  | |  |  |  |  |  |  |  |
| **worried** | .91 | .83 | | .86 | .91 | .73 | .91 | .80 | .82 | .85 |
| irritated | .05 | .07 | | .07 | .05 |  | .06 |  | .07 |  |
| sarcastic | .03 | .09 | | .07 | .04 |  | .02 |  | .10 |  |
| friendly | .01 | .01 | | .01 | .00 |  | .01 |  | .01 |  |
| 6 |  |  | |  |  |  |  |  |  |  |
| **fantasizing** | .76 | .78 | | .75 | .66 | .75 | .82 | .73 | .78 | .89 |
| aghast | .03 | .03 | | .03 | .03 |  | .03 |  | .03 |  |
| impatient | .18 | .17 | | .17 | **.27** |  | .12 |  | .16 |  |
| alarmed | .03 | .02 | | .05 | .04 |  | .03 |  | .03 |  |
| 7 |  |  | |  |  |  |  |  |  |  |
| **uneasy** | .71 | .67 | | .62 | .73 | .68 | .63 | .69 | .63 | .62 |
| apologetic | .04 | .04 | | .05 | .03 |  | .06 |  | .04 |  |
| friendly | .16 | .18 | | **.30** | .20 |  | .23 |  | .22 |  |
| dispirited | .08 | .11 | | .04 | .04 |  | .08 |  | .11 |  |
| 8 |  |  | |  |  |  |  |  |  |  |
| **despondent** | .91 | .87 | | .81 | .86 | .86 | .85 | .79 | .85 | .83 |
| relieved | .05 | .06 | | .09 | .08 |  | .08 |  | .07 |  |
| shy | .03 | .05 | | .07 | .04 |  | .06 |  | .06 |  |
| excited | .02 | .02 | | .03 | .02 |  | .01 |  | .02 |  |
| 9 |  |  | |  |  |  |  |  |  |  |
| **preoccupied** | .84 | .85 | | .80 | .69 | .82 | .89 | .88 | .85 | .81 |
| annoyed | .08 | .07 | | .09 | .20 |  | .06 |  | .09 |  |
| hostile | .02 | .06 | | .04 | .05 |  | .03 |  | .03 |  |
| horrified | .06 | .02 | | .70 | .05 |  | .02 |  | .03 |  |
| 10 |  |  | |  |  |  |  |  |  |  |
| **cautious** | .58 | .62 | | .57 | .52 | .56 | .53 | .70 | .53 | **.39** |
| insisting | **.29** | .22 | | **.33** | **.35** |  | **.32** |  | .**31** |  |
| bored | .07 | .11 | | .06 | .07 |  | .10 |  | .10 |  |
| aghast | .05 | .06 | | .03 | .06 |  | .05 |  | .05 |  |
| 11 |  |  | |  |  |  |  |  |  |  |
| **regretful** | .78 | .74 | | .78 | .87 | .76 | .75 | .77 | .68 | .77 |
| terrified | .07 | .04 | | .10 | .07 |  | .06 |  | .06 |  |
| amused | .10 | .13 | | .09 | .04 |  | .12 |  | .18 |  |
| flirtatious | .04 | .08 | | .04 | .02 |  | .06 |  | .08 |  |
| 12 |  |  | |  |  |  |  |  |  |  |
| **sceptical** | .86 | .83 | | .82 | .87 | .85 | .77 | .81 | .79 | .82 |
| indifferent | .08 | .10 | | .10 | .08 |  | .12 |  | .14 |  |
| embarrassed | .02 | .04 | | .05 | .02 |  | .04 |  | .02 |  |
| dispirited | .05 | .03 | | .03 | .03 |  | .06 |  | .05 |  |
| 13 |  |  | |  |  |  |  |  |  |  |
| **anticipating** | .69 | .74 | | .71 | .82 | .73 | .74 | .72 | .74 | .75 |
| decisive | .06 | .08 | | .08 | .04 |  | .13 |  | .09 |  |
| threatening | .06 | .03 | | .06 | .02 |  | .04 |  | .04 |  |
| shy | .19 | .15 | | .15 | .13 |  | .09 |  | .13 |  |
| 14 |  |  | |  |  |  |  |  |  |  |
| **accusing** | .75 | .82 | | .69 | .74 | .79 | .80 | .85 | .76 | .67 |
| irritated | .13 | .11 | | .14 | .17 |  | .08 |  | .15 |  |
| disappointed | .08 | .05 | | .12 | .02 |  | .09 |  | .05 |  |
| depressed | .04 | .02 | | .06 | .07 |  | .03 |  | .03 |  |
| 15 |  |  | |  |  |  |  |  |  |  |
| **contemplative** | .81 | .74 | | .79 | .78 | .76 | .76 | .82 | .72 | .68 |
| flustered | .02 | .06 | | .07 | .03 |  | .05 |  | .03 |  |
| encouraging | .07 | .10 | | .08 | .06 |  | .05 |  | .10 |  |
| amused | .10 | .10 | | .06 | .13 |  | .14 |  | .15 |  |
| 16 |  |  | |  |  |  |  |  |  |  |
| **thoughtful** | .77 | .72 | | .81 | .75 | .68 | .75 | .84 | .70 | .69 |
| irritated | .06 | .06 | | .05 | .05 |  | .06 |  | .07 |  |
| encouraging | .05 | .04 | | .07 | .06 |  | .08 |  | .05 |  |
| sympathetic | .12 | .18 | | .08 | .14 |  | .12 |  | .17 |  |
| 17 |  |  | |  |  |  |  |  |  |  |
| **doubtful** | .55 | .58 | | .62 | .66 | .60 | **.45** | .59 | **.47** | .50 |
| affectionate | **.26** | **.25** | | **.27** | .22 |  | **.34** |  | **.31** |  |
| playful | .16 | .11 | | .08 | .08 |  | .17 |  | .17 |  |
| aghast | .03 | .06 | | .03 | .03 |  | .04 |  | .05 |  |
| 18 |  |  | |  |  |  |  |  |  |  |
| **decisive** | .66 | .69 | | .68 | .68 | .68 | .65 | .76 | .65 | .56 |
| amused | .18 | .11 | | .18 | .11 |  | .22 |  | .21 |  |
| aghast | .04 | .07 | | .05 | .03 |  | .04 |  | .05 |  |
| bored | .12 | .13 | | .09 | .18 |  | .10 |  | .10 |  |
| 19 |  |  | |  |  |  |  |  |  |  |
| **tentative** | .68 | .64 | | **.49** | .55 | .68 | .63 | .67 | .64 | .52 |
| arrogant | .08 | .09 | | .10 | .08 |  | .07 |  | .07 |  |
| grateful | .18 | .19 | | **.29** | **.26** |  | .24 |  | .19 |  |
| sarcastic | .06 | .09 | | .12 | .10 |  | .06 |  | .09 |  |
| 20 |  |  | |  |  |  |  |  |  |  |
| **friendly** | .84 | .87 | | .84 | .75 | .83 | .84 | .91 | .86 | .81 |
| dominant | .09 | .05 | | .11 | .15 |  | .06 |  | .07 |  |
| guilty | .06 | .07 | | .04 | .10 |  | .08 |  | .06 |  |
| horrified | .01 | .00 | | .01 | .00 |  | .03 |  | .01 |  |
| 21 |  |  | |  |  |  |  |  |  |  |
| **fantasizing** | .91 | .80 | | .86 | .81 | .86 | .85 | .84 | .83 | .86 |
| embarrassed | .05 | .13 | | .06 | .11 |  | .08 |  | .10 |  |
| confused | .03 | .06 | | .07 | .07 |  | .05 |  | .05 |  |
| panicked | .00 | .01 | | .01 | .00 |  | .02 |  | .01 |  |
| 22 |  |  | |  |  |  |  |  |  |  |
| **preoccupied** | .80 | .74 | | .80 | .76 | .73 | .73 | .87 | .73 | .69 |
| grateful | .12 | .01 | | .05 | .01 |  | .02 |  | .03 |  |
| insisting | .05 | .04 | | .09 | .04 |  | .10 |  | .05 |  |
| imploring | .13 | .20 | | .06 | .19 |  | .15 |  | .19 |  |
| 23 |  |  | |  |  |  |  |  |  |  |
| **defiant** | .59 | .61 | | .55 | .52 | .61 | .56 | .66 | .57 | **.32** |
| contented | .10 | .06 | | .09 | .07 |  | .13 |  | .08 |  |
| apologetic | .06 | .07 | | .12 | .14 |  | .10 |  | .07 |  |
| curious | .25^i^ | **.25** | | .24 | **.28** |  | .22 |  | **.28** |  |
| 24 |  |  | |  |  |  |  |  |  |  |
| **pensive** | .86 | .72 | | .84 | .69 | .70 | .78 | .79 | .74 | 0.57 |
| irritated | .06 | .12 | | .08 | .20 |  | .13 |  | .13 |  |
| excited | .03 | .03 | | .04 | .02 |  | .02 |  | .04 |  |
| hostile | .05 | .13 | | .05 | .09 |  | .07 |  | .09 |  |
| 25 |  |  | |  |  |  |  |  |  |  |
| **interested** | .76 | .65 | | .64 | .61 | .66 | .71 | .67 | .74 | .65 |
| panicked | .02 | .07 | | .06 | .08 |  | .09 |  | .05 |  |
| incredulous | .15 | .20 | | .22 | .22 |  | .13 |  | .15 |  |
| despondent | .07 | .09 | | .08 | .09 |  | .07 |  | .07 |  |
| 26 |  |  | |  |  |  |  |  |  |  |
| **hostile** | .70 | .74 | | .71 | .69 | .79 | .69 | .72 | .66 | .67 |
| alarmed | .08 | .07 | | .08 | .05 |  | .10 |  | .07 |  |
| shy | .05 | .06 | | .07 | .12 |  | .06 |  | .08 |  |
| anxious | .17 | .13 | | .14 | .14 |  | .15 |  | .19 |  |
| 27 |  |  | |  |  |  |  |  |  |  |
| **cautious** | .81 | .75 | | .80 | .79 | .75 | .77 | .78 | .71 | .77 |
| joking | .01 | .01 | | .04 | .01 |  | .03 |  | .02 |  |
| arrogant | .09 | .13 | | .09 | .14 |  | .07 |  | .14 |  |
| reassuring | .09 | .10 | | .08 | .06 |  | .13 |  | .14 |  |
| 28 |  |  | |  |  |  |  |  |  |  |
| **interested** | .69 | .70 | | .67 | .82 | .74 | .72 | .77 | .64 | .62 |
| joking | .01 | .03 | | .03 | .02 |  | .05 |  | .03 |  |
| affectionate | **.26** | .14 | | .24 | .11 |  | .18 |  | .19 |  |
| contented | .05 | .13 | | .05 | .05 |  | .06 |  | .14 |  |
| 29 |  |  | |  |  |  |  |  |  |  |
| **reflective** | .78 | .59 | | .72 | .56 | .55 | .69 | .66 | .68 | **.44** |
| impatient | .09 | .18 | | .12 | .20 |  | .14 |  | .12 |  |
| aghast | .02 | .03 | | .04 | .01 |  | .05 |  | .02 |  |
| irritated | .11 | .20 | | .11 | .23 |  | .12 |  | .18 |  |
| 30 |  |  | |  |  |  |  |  |  |  |
| **flirtatious** | .95 | .87 | | .87 | .81 | .81 | .88 | .85 | .87 | .79 |
| grateful | .01 | .03 | | .05 | .01 |  | .04 |  | .04 |  |
| hostile | .03 | .06 | | .07 | .12 |  | .04 |  | .05 |  |
| disappointed | .01 | .04 | | .02 | .05 |  | .04 |  | .04 |  |
| 31 |  |  | |  |  |  |  |  |  |  |
| **confident** | .81 | .59 | | .75 | .67 | .67 | .70 | .68 | .64 | .67 |
| ashamed | .03 | .11 | | .05 | .07 |  | .07 |  | .07 |  |
| joking | .06 | .09 | | .13 | .13 |  | .08 |  | .07 |  |
| dispirited | .10 | .21 | | .07 | .13 |  | .15 |  | .22 |  |
| 32 |  |  | |  |  |  |  |  |  |  |
| **serious** | .82 | .79 | | .78 | .79 | .80 | .75 | .76 | .75 | .78 |
| ashamed | .02 | .05 | | .06 | .03 |  | .03 |  | .03 |  |
| bewildered | .08 | .11 | | .07 | .09 |  | .12 |  | .13 |  |
| alarmed | .08 | .05 | | .09 | .09 |  | .10 |  | .09 |  |
| 33 |  |  | |  |  |  |  |  |  |  |
| **concerned** | .72 | .71 | | .72 | .71 | .76 | .73 | .69 | .74 | .73 |
| embarrassed | .03 | .05 | | .05 | .06 |  | .07 |  | .05 |  |
| guilty | .14 | .21 | | .13 | .19 |  | .15 |  | .16 |  |
| fantasizing | .11 | .03 | | .10 | .05 |  | .04 |  | .05 |  |
| 34 |  |  | |  |  |  |  |  |  |  |
| **distrustful** | .75 | .71 | | .70 | .79 | .73 | .63 | .73 | .62 | .56 |
| aghast | .06 | .05 | | .05 | .03 |  | .08 |  | .07 |  |
| baffled | .14 | .18 | | .23 | .15 |  | .24 |  | .22 |  |
| terrified | .05 | .07 | | .02 | .03 |  | .05 |  | .08 |  |
| 35 |  |  | |  |  |  |  |  |  |  |
| **nervous** | .50 | .75 | | **.48** | .68 | .73 | **.47** | .75 | .57 | .65 |
| puzzled | .07 | .09 | | .10 | .11 |  | .12 |  | .10 |  |
| insisting | .19 | .07 | | .25^i^ | .12 |  | .19 |  | .12 |  |
| contemplative | .24 | .09 | | .18 | .10 |  | .22 |  | .20 |  |
| 36 |  |  | |  |  |  |  |  |  |  |
| **suspicious** | .93 | .88 | | .86 | .91 | .91 | .88 | .85 | .83 | .89 |
| ashamed | .01 | .01 | | .03 | .01 |  | .03 |  | .02 |  |
| nervous | .03 | .02 | | .06 | .03 |  | .05 |  | .05 |  |
| indecisive | .04 | .08 | | .04 | .06 |  | .05 |  | .11 |  |
| Number of items failing original retention criteria | | |  |  |  |  |  |  |  |  |
| < 50% correct | 0 | 0 | | 2 | 0 | 0 | 2 | 0 | 1 | 3 |
| > 25% same incorrect | 3 | 2 | | 4 | 4 | N/A | 2 | N/A | 4 | N/A |

*Note.* In the “RMET item” column, the target response is presented in bold font. In the remaining columns, target responses with a response rate of ≤ .50 and foil responses with a response rate of ≥ .25 are presented in a bold font. Where datasets coded RMET scores as correct/incorrect rather than specifying which foil item was selected for incorrect responses, response rates are only provided for the target response. ^i^This is not highlighted because the actual value was less than .25 before being rounded.

**Table S3**

*Standardised Factor Loadings for Single-factor CFA Model*

| **RMET item** | **Almaatouq** | **Dodell-Feder** | **Floyd** | **Kidd** | **Nahal** | **Panero** | **Pearce** | **Schmalor** | **Vonk** |
| --- | --- | --- | --- | --- | --- | --- | --- | --- | --- |
| 1 | .230 | .186 | .293 | .143^*^ | .185 | .489 | .155^*^ | .129^**^ | .181 |
| 2 | .321 | .232 | .233 | .238 | .232 | .312 | .300 | .331 | .256 |
| 3 | .278 | .413 | .335 | .278 | .297 | .474 | .270 | .279 | .322 |
| 4 | .356 | .212 | .529 | .225 | .242 | .438 | .137^*^ | .370 | **.098** |
| 5 | .357 | .279 | .673 | .267 | .274 | .692 | .356 | .457 | .227 |
| 6 | .317 | .312 | .313 | .275 | .357 | .513 | .147^*^ | .239 | .173^*^ |
| 7 | .306 | .249 | .498 | .202 | .321 | .422 | .**115** | .290 | .105^*^ |
| 8 | .638 | .389 | .725 | .243 | .343 | .645 | .326 | .397 | .455 |
| 9 | .420 | .475 | .694 | .327 | .504 | .687 | .302 | .253 | .444 |
| 10 | .321 | .260 | .347 | .163 | .166 | .308 | .304 | .280 | .155^**^ |
| 11 | .347 | .325 | .571 | .325 | .319 | .618 | .337 | .403 | .447 |
| 12 | .360 | .432 | .615 | .393 | .547 | .609 | .283 | .430 | .261 |
| 13 | .355 | .397 | .377 | .192 | .399 | .649 | .250 | .285 | .326 |
| 14 | .336 | .480 | .619 | .253 | .401 | .644 | .399 | .395 | .336 |
| 15 | .442 | .517 | .712 | .260 | .507 | .626 | .484 | .487 | .340 |
| 16 | .428 | .349 | .416 | .368 | .219 | .542 | .263 | .360 | .257 |
| 17 | .303 | .157 | .465 | **.096** | .208 | .358 | .212 | .246 | .192 |
| 18 | .366 | .344 | .474 | .240 | .376 | .335 | .286 | .426 | .287 |
| 19 | .255 | .385 | .490 | .302 | .491 | .208 | .368 | .272 | .347 |
| 20 | .310 | .443 | .386 | .214 | .365 | .627 | .500 | .384 | .350 |
| 21 | .412 | .289 | .546 | .373 | .493 | .453 | .328 | .405 | .495 |
| 22 | .520 | .447 | .747 | .292 | .321 | .682 | .387 | .502 | .373 |
| 23 | .282 | .318 | .414 | .151^**^ | .362 | .549 | .203 | .476 | .**101** |
| 24 | .519 | .462 | .604 | .163^**^ | .444 | .655 | .293 | .427 | .421 |
| 25 | .230 | .145 | .405 | .210 | **.087** | .355 | **.047** | .235 | .315 |
| 26 | .176 | .378 | .454 | .224 | .548 | .464 | .318 | .366 | .249 |
| 27 | .518 | .438 | .540 | .388 | .390 | .488 | .283 | .443 | .370 |
| 28 | .395 | .459 | .472 | .354 | .471 | .525 | .398 | .457 | .415 |
| 29 | .266 | .211 | .531 | .131^**^ | .174 | .518 | **.057** | .145^**^ | .236 |
| 30 | .761 | .422 | .591 | .170^**^ | .441 | .671 | .315 | .504 | .336 |
| 31 | .305 | .209 | .294 | .339 | .160 | .328 | .224 | .114^*^ | .277 |
| 32 | .359 | .397 | .543 | .364 | .300 | .509 | .341 | .574 | .565 |
| 33 | .303 | .270 | .630 | .234 | .307 | .542 | .211 | .248 | .530 |
| 34 | .472 | .399 | .590 | .331 | .351 | .528 | .359 | .407 | .225 |
| 35 | .213 | .332 | .223 | .157^**^ | .327 | .361 | .323 | .345 | .225 |
| 36 | .343 | .386 | .709 | .410 | .457 | .743 | .266 | .648 | .584 |
| Loadings < .3 | 8 | 12 | 3 | 24 | 11 | 1 | 18 | 13 | 17 |

*Note.* p ≤ .001 unless otherwise specified. ^*^ p ≤ .05; ^**^ p ≤ .01; non-significant factor loadings (at alpha = .05) are in bold.

**Table S4**

*Table of Factor Loadings for Three-factor Valence-based CFA Model*

|  | **Almaatouq** | **Dodell-Feder** | **Floyd** | **Kidd** | **Nahal** | **Panero** | **Pearce** | **Schmalor** | **Vonk** |
| --- | --- | --- | --- | --- | --- | --- | --- | --- | --- |
| Factor 1: Positive |  |  |  |  |  |  |  |  |  |
| 1 Playful | .302 | .244 | .348 | .165^*^ | .259 | .563 | .196^**^ | .155^**^ | .225 |
| 6 Fantasising | .404 | .400 | .370 | .310 | .456 | .587 | .204^**^ | .304 | .202^*^ |
| 16 Thoughtful | .497 | .429 | .484 | .416 | .256 | .613 | .278 | .434 | .305 |
| 20 Friendly | .373 | .561 | .455 | .253 | .454 | .717 | .593 | .474 | .447 |
| 21 fantasising | .483 | .367 | .643 | .422 | .597 | .521 | .432 | .504 | .643 |
| 25 Interested | .290 | .198 | .482 | .261 | .137^**^ | .411 | **.101** | .306 | .426 |
| 30 Flirtatious | .917 | .540 | .702 | .200 | .543 | .767 | .417 | .633 | .471 |
| 31 Confident | .373 | .280 | .341 | .394 | .209 | .382 | .309 | .166^**^ | .400 |
| Factor 2: Negative |  |  |  |  |  |  |  |  |  |
| 2 Upset | .339 | .242 | .237 | .258 | .237 | .324 | .308 | .339 | .265 |
| 5 Worried | .387 | .293 | .682 | .279 | .283 | .715 | .386 | .470 | .234 |
| 11 Regretful | .378 | .343 | .579 | .352 | .333 | .640 | .357 | .420 | .465 |
| 14 Accusing | .349 | .504 | .627 | .272 | .413 | .668 | .428 | .404 | .346 |
| 17 Doubtful | .332 | .171 | .474 | .115^*^ | .222 | .378 | .225 | .257 | .208 |
| 22 Preoccupied | .558 | .465 | .758 | .306 | .329 | .704 | .417 | .516 | .383 |
| 23 Defiant | .307 | .338 | .421 | .166 | .372 | .571 | .227 | .492 | **.106** |
| 26 Hostile | .184 | .397 | .460 | .227 | .567 | .482 | .338 | .397 | .257 |
| 27 Cautious | .557 | .458 | .548 | .421 | .404 | .506 | .298 | .455 | .387 |
| 34 Distrustful | .513 | .420 | .599 | .358 | .364 | .546 | .394 | .421 | .237 |
| 35 Nervous | .234 | .351 | .229 | .188 | .341 | .378 | .354 | .359 | .232 |
| 36 Suspicious | .365 | .403 | .720 | .446 | .472 | .768 | .297 | .665 | .605 |
| Factor 3: Neutral |  |  |  |  |  |  |  |  |  |
| 3 Desire | .279 | .416 | .336 | .274 | .299 | .475 | .276 | .282 | .327 |
| 4 Insisting | .357 | .216 | .532 | .222 | .245 | .439 | .136^*^ | .375 | **.103** |
| 7 Uneasy | .307 | .253 | .501 | .197 | .325 | .423 | **.108** | .294 | .116^*^ |
| 8 Despondent | .640 | .393 | .729 | .243 | .345 | .647 | .325 | .401 | .473 |
| 9 Preoccupied | .421 | .481 | .698 | .323 | .508 | .688 | .308 | .257 | .456 |
| 10 Cautious | .322 | .264 | .349 | .161 | .168 | .308 | .304 | .283 | .165 |
| 12 Sceptical | .361 | .438 | .618 | .390 | .551 | .611 | .282 | .435 | .269 |
| 13 Anticipating | .356 | .402 | .379 | .192 | .402 | .650 | .250 | .288 | .332 |
| 15 Contemplative | .444 | .525 | .716 | .258 | .511 | .628 | .488 | .493 | .357 |
| 18 Decisive | .367 | .349 | .477 | .237 | .380 | .336 | .292 | .432 | .294 |
| 19 Tentative | .255 | .391 | .493 | .298 | .494 | .208 | .365 | .274 | .359 |
| 24 Pensive | .521 | .469 | .607 | .161^**^ | .446 | .657 | .303 | .432 | .436 |
| 28 Interested | .396 | .465 | .475 | .248 | .475 | .526 | .395 | .481 | .424 |
| 29 Reflective | .266 | .212 | .533 | .130^**^ | .174 | .518 | .**064** | .147 | .236 |
| 32 Serious | .359 | .401 | .545 | .361 | .301 | .510 | .354 | .582 | .576 |
| 33 Concerned | .304 | .273 | .634 | .231 | .309 | .542 | .217 | .251 | .547 |
| Factor correlations(SE) |  |  |  |  |  |  |  |  |  |
| Positive~~Negative | .584(.056) | .619(.022) | .774(.042) | .621(.089) | .665(.060) | .721(.050) | .556(.101) | .673(.054) | .641(.077) |
| Positive~~Neutral | .809(.048) | .731(.019) | .813(.035) | .883(.084) | .751(.051) | .861(.038) | .819(.104) | .776(.057) | .615(.062) |
| Negative~~Neutral | .930(.038) | .929(.013) | .977(.016) | .953(.084) | .960(.036) | .965(.024) | .884(.072) | .943(.032) | .936(.050) |
| Loadings < .3 | 6 | 11 | 2 | 24 | 11 | 1 | 16 | 10 | 15 |

*Note.* p ≤ .001 unless otherwise specified. ^*^ p ≤ .05; ^**^ p ≤ .01; non-significant factor loadings (at alpha = .05) are in bold. SE = standard error.

**Figure S1**

*Scree Plots for Split Datasets*

**
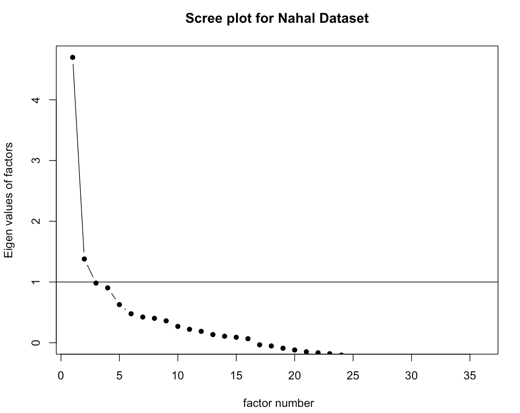
**
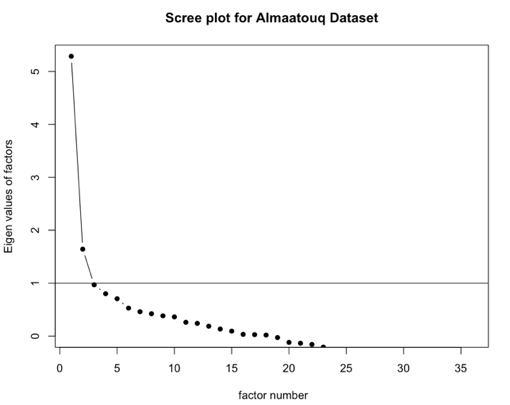

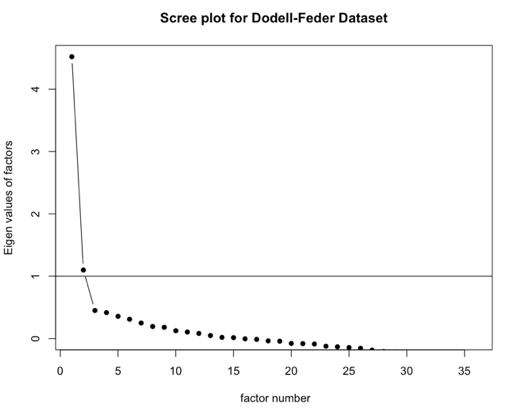
**
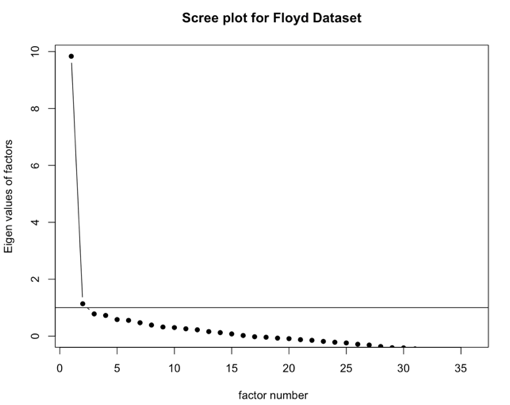

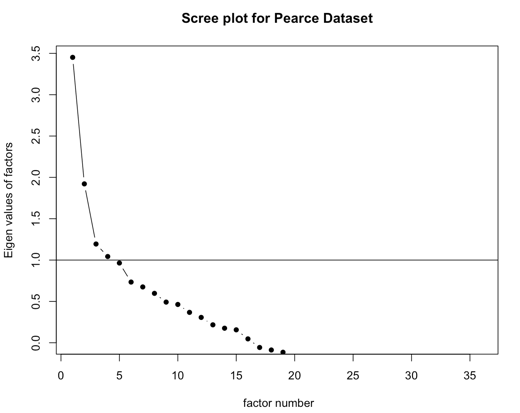

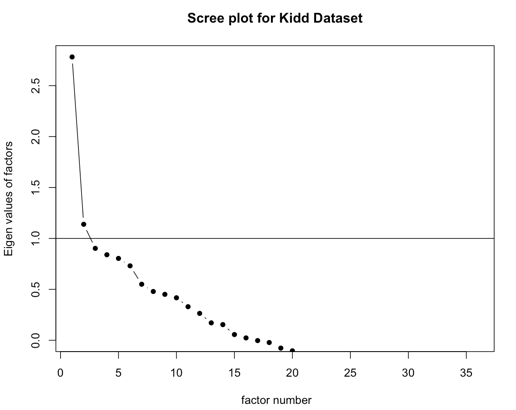
**

**
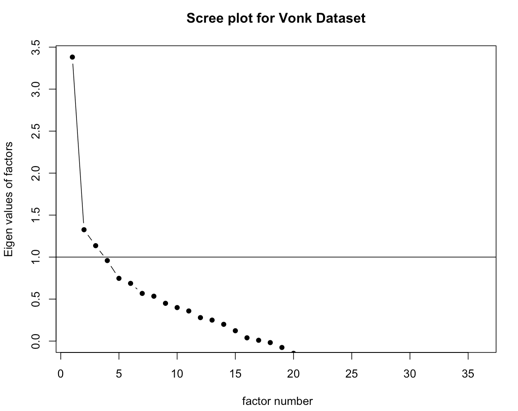

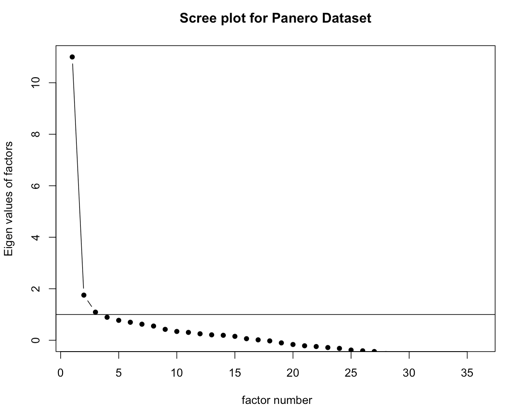

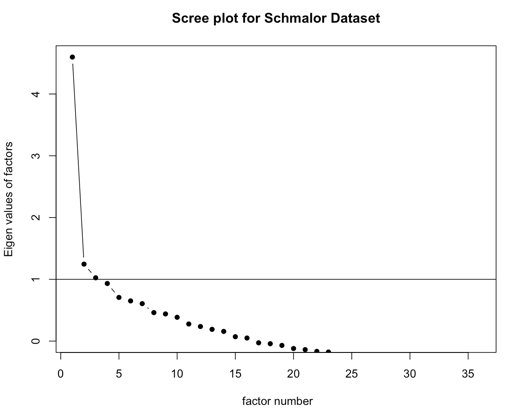
**

**Figure S2**


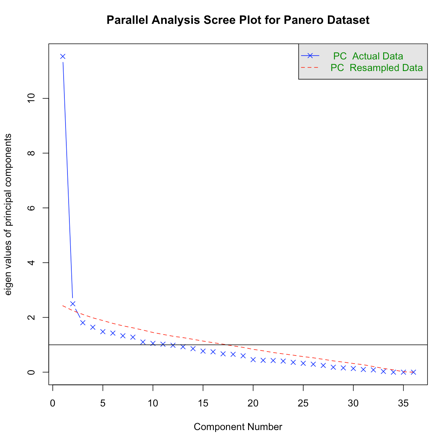

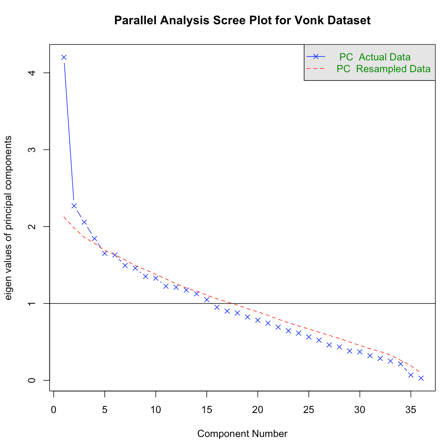

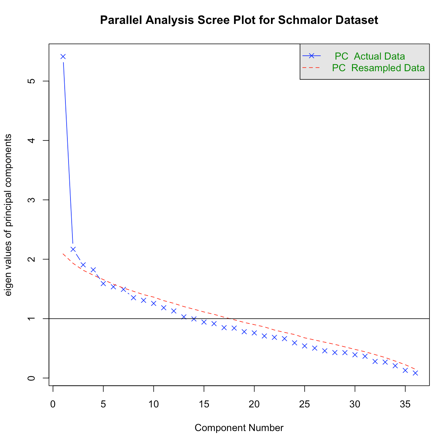

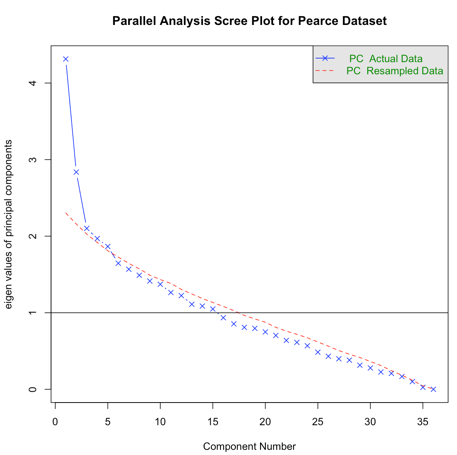
*
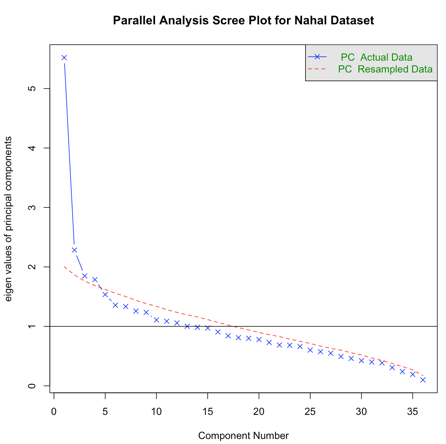

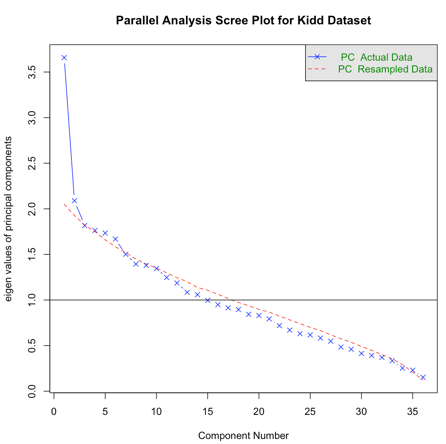
*
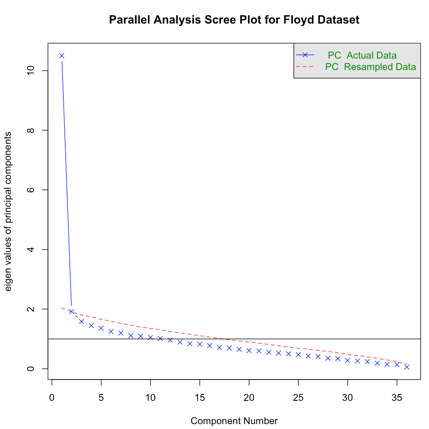
**
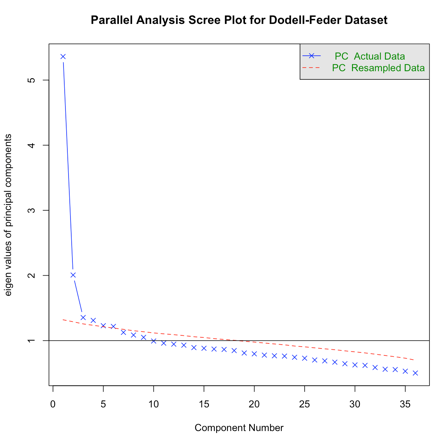
**
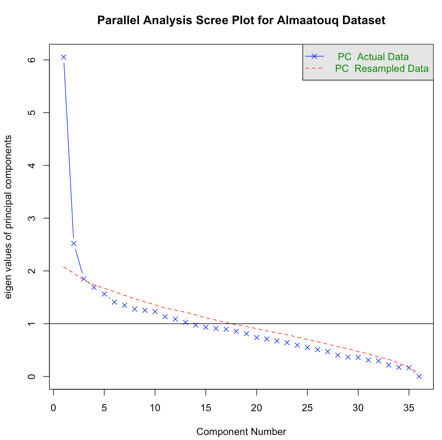
*Parallel Analysis Scree Plots for Split Datasets*

**Table S5**

*Factor Loadings for Two-Factor EFA Models Using Oblimin Rotation*

| **Dataset** | **Almaatouq** |  | | **Dodell** |  | **Nahal** | |  | | **Panero** |  | | **Pearce** |  |
| --- | --- | --- | --- | --- | --- | --- | --- | --- | --- | --- | --- | --- | --- | --- |
| Item - target | Negative | Positive | | Negative | Positive | Negative | | Positive | | Negative | Positive | | Negative | Positive |
| 1 - playful | -0.010 | 0.273 | | -0.024 | **0.335** | -0.072 | | **0.362** | | -0.009 | **0.520** | | 0.088 | 0.141 |
| 2 - upset | **0.350** | 0.020 | | 0.162 | 0.099 | 0.136 | | 0.203 | | 0.220 | 0.157 | | 0.046 | **0.526** |
| 3 - desire | 0.018 | **0.400** | | 0.109 | **0.385** | 0.061 | | **0.407** | | -0.091 | **0.667** | | 0.067 | **0.449** |
| 4 - insisting | **0.441** | 0.004 | | **0.409** | -0.206 | **0.365** | | -0.110 | | **0.495** | 0.044 | | 0.124 | 0.064 |
| 5 - worried | **0.464** | -0.030 | | 0.242 | 0.066 | 0.222 | | 0.101 | | **0.395** | **0.442** | | **0.448** | 0.036 |
| 6 - fantasizing | -0.161 | **0.670** | | -0.081 | **0.487** | 0.034 | | **0.540** | | -0.137 | **0.679** | | -0.106 | 0.227 |
| 7 - uneasy | **0.511** | -0.176 | | **0.416** | -0.172 | **0.490** | | -0.138 | | **0.628** | -0.075 | | **0.319** | **-0.350** |
| 8 - despondent | **0.555** | 0.276 | | **0.308** | 0.181 | 0.247 | | 0.207 | | 0.297 | **0.444** | | **0.352** | 0.171 |
| 9 - preoccupied | 0.099 | **0.560** | | 0.272 | **0.300** | 0.289 | | **0.329** | | 0.296 | **0.557** | | **0.375** | 0.068 |
| 10 - cautious | **0.324** | 0.029 | | **0.419** | -0.161 | 0.257 | | -0.147 | | 0.216 | 0.169 | | **0.320** | -0.067 |
| 11 - regretful | **0.476** | -0.128 | | **0.391** | -0.031 | **0.500** | | -0.156 | | 0.299 | **0.442** | | **0.376** | 0.170 |
| 12 - sceptical | **0.330** | 0.092 | | **0.433** | 0.029 | **0.403** | | 0.165 | | **0.473** | 0.247 | | **0.457** | -0.080 |
| 13 - anticipating | 0.221 | 0.214 | | 0.283 | 0.165 | 0.256 | | 0.206 | | **0.492** | **0.321** | | **0.333** | -0.113 |
| 14 - accusing | 0.198 | 0.249 | | **0.449** | 0.080 | 0.198 | | **0.311** | | **0.699** | 0.088 | | 0.207 | **0.389** |
| 15 - contemplative | 0.272 | 0.296 | | **0.430** | 0.176 | **0.413** | | 0.203 | | **0.376** | 0.271 | | **0.496** | -0.050 |
| 16 - thoughtful | 0.289 | **0.394** | | 0.187 | 0.211 | 0.236 | | 0.105 | | 0.109 | **0.511** | | 0.200 | 0.057 |
| 17 - doubtful | **0.536** | -0.167 | | **0.370** | -0.230 | **0.476** | | -0.291 | | **0.651** | -0.167 | | **0.319** | -0.265 |
| 18 - decisive | **0.301** | 0.118 | | **0.354** | 0.039 | **0.361** | | 0.057 | | 0.246 | 0.152 | | 0.158 | **0.327** |
| 19 - tentative | 0.227 | 0.046 | | **0.341** | 0.065 | **0.373** | | 0.227 | | 0.273 | 0.010 | | 0.285 | -0.007 |
| 20 - friendly | 0.199 | 0.213 | | 0.078 | **0.514** | 0.058 | | **0.352** | | 0.163 | **0.601** | | 0.278 | **0.374** |
| 21 - fantasizing | 0.080 | **0.370** | | 0.010 | **0.382** | 0.214 | | **0.353** | | -0.192 | **0.650** | | 0.011 | **0.458** |
| 22 - preoccupied | **0.370** | 0.252 | | **0.303** | 0.211 | 0.248 | | 0.169 | | **0.372** | **0.461** | | **0.418** | 0.109 |
| 23 - defiant | **0.411** | -0.158 | | **0.331** | -0.015 | **0.416** | | 0.050 | | **0.630** | 0.090 | | 0.249 | 0.055 |
| 24 - pensive | 0.251 | **0.300** | | **0.324** | 0.219 | **0.347** | | 0.195 | | 0.259 | **0.549** | | -0.087 | **0.548** |
| 25 - interested | -0.192 | **0.448** | | -0.117 | **0.353** | -0.235 | | **0.463** | | -0.151 | **0.632** | | -0.085 | **0.318** |
| 26 - hostile | 0.231 | -0.058 | | **0.314** | 0.120 | **0.409** | | 0.265 | | 0.279 | 0.217 | | 0.273 | 0.226 |
| 27 - cautious | **0.505** | 0.105 | | **0.321** | 0.196 | **0.381** | | 0.129 | | **0.540** | 0.016 | | **0.420** | -0.025 |
| 28 - interested | **0.345** | 0.084 | | **0.394** | 0.125 | **0.497** | | 0.142 | | **0.348** | 0.209 | | **0.520** | 0.026 |
| 29 - reflective | -0.133 | **0.560** | | 0.001 | 0.269 | -0.005 | | 0.180 | | -0.024 | **0.664** | | 0.005 | 0.165 |
| 30 - flirtatious | **0.354** | **0.588** | | 0.116 | **0.455** | 0.065 | | **0.565** | | 0.112 | **0.757** | | 0.056 | **0.448** |
| 31 - confident | 0.011 | **0.388** | | -0.040 | **0.336** | -0.011 | | 0.279 | | -0.244 | **0.620** | | -0.053 | **0.459** |
| 32 - serious | 0.257 | 0.172 | | 0.144 | **0.352** | 0.091 | | 0.212 | | 0.245 | **0.368** | | -0.028 | **0.461** |
| 33 - concerned | 0.263 | 0.131 | | 0.061 | 0.244 | 0.211 | | 0.115 | | 0.221 | **0.428** | | 0.152 | 0.090 |
| 34 - distrustful | **0.466** | 0.017 | | **0.333** | 0.117 | 0.299 | | 0.133 | | **0.467** | 0.218 | | **0.455** | -0.029 |
| 35 - nervous | **0.330** | -0.068 | | **0.389** | -0.017 | **0.460** | | -0.073 | | **0.665** | -0.133 | | **0.396** | 0.041 |
| 36 - suspicious | 0.139 | 0.211 | | **0.314** | 0.092 | **0.495** | | 0.003 | | **0.379** | **0.469** | | 0.292 | 0.052 |
| Proportion variance | .110 | .090 | | .092 | .067 | .072 | | .104 | | .163 | .204 | | .086 | .073 |
| Total cumulative variance | .200 | | .159 | | | | .175 | | .367 | | | .158 | | |
| Items with no loading > .30 | 10 | | 6 | | | | 12 | | 5 | | | 10 | | |

*Note.* Factor loadings > .30 are presented in bold font.

**Table S6**

*Fit Indices for Two-factor EFA Models*

| **Dataset** |  | **Chi-square (χ²)** | **CFI** | **TLI** | **RMSEA (90% CI)** | **SRMR** |
| --- | --- | --- | --- | --- | --- | --- |
| Almaatouq | WLSMV | χ²(559, n = 591) = 612, p = .061 | .951 | .945 | .013(.000, .019) | .072 |
|  | ML |  | .458^*^ | .389^*^ | .109(.102, .117)^*^ |  |
| Dodell-Feder | WLSMV | χ²(559, n = 4634) = 1262, p < .001 | .935 | .927 | .016(.015, .018) | .035 |
|  | ML |  | .859 | .841 | .032(.030, .035) |  |
| Nahal | WLSMV | χ²(559, n = 584) = 601, p = .104 | .960 | .955 | .020(.015, .025) | .067 |
|  | ML |  | .738 | .705 | .060(.052, .068) |  |
| Panero | WLSMV | χ²(559, n = 279) = 617 p = .044 | .971 | .967 | .019 (.003, .028) | .081 |
|  | ML |  | .470^*^ | .403^*^ | .175(.165, .185)^*^ |  |
| Pearce | WLSMV | χ²(559, n = 354) = 611 p = .062 | .848 | .828 | .016(.000, .024) | .092 |
|  | ML |  | .257^*^ | .163^*^ | .134(.125, .143)^*^ |  |

*Note.* *The tetrachoric correlation matrix was not positive definite, so these ML-based fit indices values might be less accurate.

**Table S7**

*Standardised Factor Loadings for Three-factor EFA Models with Default Geomin Rotation (as preregistered)*

| **Dataset** | **Almaatouq** | | | **Dodell-Feder** | | | **Nahal** | | | **Panero** | | | **Pearce** | | |
| --- | --- | --- | --- | --- | --- | --- | --- | --- | --- | --- | --- | --- | --- | --- | --- |
| RMET item | f1 | f2 | f3 | f1 | f2 | f3 | f1 | f2 | f3 | f1 | f2 | f3 | f1 | f2 | f3 |
| 1 - playful | 0.084 | 0.033 | 0.267 | **0.624** | 0.019 | -0.139 | 0.144 | **0.403** | -0.115 | 0.154 | 0.215 | **0.343** | -0.032 | 0.083 | 0.147 |
| 2 - upset | 0.131 | **0.584** | -0.025 | 0.013 | 0.189 | 0.079 | 0.177 | 0.101 | 0.116 | 0.168 | **0.422** | 0.171 | -0.060 | 0.028 | **0.544** |
| 3 - desire | 0.009 | -0.027 | **0.423** | 0.090 | 0.168 | **0.326** | **0.598** | -0.081 | 0.016 | **0.543** | -0.018 | 0.266 | **0.628** | 0.004 | **0.441** |
| 4 - insisting | **0.513** | -0.003 | 0.177 | 0.248 | **0.405** | -0.099 | 0.023 | -0.180 | **0.371** | **0.422** | 0.286 | **0.353** | **0.482** | 0.104 | 0.010 |
| 5 - worried | 0.275 | 0.272 | 0.070 | 0.034 | 0.270 | 0.044 | 0.110 | 0.031 | 0.210 | **0.494** | **0.369** | 0.020 | 0.008 | **0.444** | 0.034 |
| 6 - fantasizing | 0.276 | 0.001 | **0.620** | **0.397** | -0.011 | 0.198 | **0.475** | 0.248 | -0.012 | **0.489** | -0.023 | **0.317** | 0.093 | -0.122 | 0.228 |
| 7 - uneasy | **0.337** | 0.287 | -0.067 | **0.338** | **0.424** | 0.010 | 0.034 | -0.246 | **0.508** | 0.042 | **0.568** | 0.244 | 0.181 | **0.336** | **-0.391** |
| 8 - despondent | 0.279 | **0.330** | **0.402** | 0.010 | **0.356** | 0.137 | 0.009 | **0.359** | 0.218 | **0.402** | **0.328** | 0.060 | 0.055 | **0.341** | 0.169 |
| 9 - preoccupied | 0.038 | 0.074 | **0.590** | 0.013 | **0.332** | 0.260 | 0.161 | **0.335** | 0.251 | **0.505** | **0.330** | 0.102 | **-0.461** | **0.400** | 0.107 |
| 10 - cautious | 0.160 | 0.215 | 0.092 | 0.247 | **0.423** | -0.053 | 0.072 | -0.129 | 0.269 | 0.294 | 0.145 | 0.081 | 0.154 | **0.319** | -0.087 |
| 11 - regretful | **0.346** | 0.227 | -0.013 | 0.029 | **0.420** | -0.108 | 0.035 | -0.173 | **0.508** | **0.462** | 0.293 | 0.024 | -0.008 | **0.370** | 0.171 |
| 12 - sceptical | 0.195 | 0.177 | 0.170 | 0.092 | **0.470** | 0.008 | **0.337** | -0.152 | **0.396** | 0.168 | **0.530** | 0.009 | 0.105 | **0.458** | -0.097 |
| 13 - anticipating | 0.064 | 0.168 | 0.254 | 0.126 | **0.338** | -0.013 | 0.292 | -0.033 | 0.240 | **0.365** | **0.476** | 0.069 | -0.133 | **0.342** | -0.103 |
| 14 - accusing | **0.311** | -0.133 | **0.381** | 0.057 | **0.494** | 0.031 | **0.392** | 0.007 | 0.172 | 0.043 | **0.736** | 0.120 | 0.151 | 0.186 | **0.379** |
| 15 - contemplative | 0.243 | 0.022 | **0.409** | 0.045 | **0.492** | 0.044 | 0.187 | 0.106 | **0.389** | 0.055 | **0.516** | 0.097 | 0.030 | **0.497** | -0.059 |
| 16 - thoughtful | 0.037 | **0.329** | **0.418** | 0.099 | 0.235 | 0.093 | 0.073 | 0.274 | 0.221 | 0.060 | **0.386** | **0.362** | -0.049 | 0.201 | 0.060 |
| 17 - doubtful | **0.337** | **0.308** | -0.051 | 0.022 | **0.403** | **-0.419** | **0.371** | 0.019 | **0.505** | 0.005 | **0.564** | 0.296 | -0.046 | **0.330** | -0.268 |
| 18 - decisive | 0.161 | 0.176 | 0.184 | 0.067 | **0.385** | 0.019 | 0.125 | -0.050 | **0.354** | **0.438** | 0.078 | 0.189 | 0.158 | 0.139 | **0.316** |
| 19 - tentative | 0.011 | 0.260 | 0.056 | 0.047 | **0.378** | 0.026 | 0.166 | 0.172 | **0.345** | 0.149 | **0.375** | 0.025 | -0.273 | **0.303** | 0.015 |
| 20 - friendly | 0.088 | 0.300 | 0.208 | 0.248 | 0.164 | **0.318** | 0.107 | **0.433** | 0.017 | 0.034 | **0.551** | **0.490** | -0.006 | 0.264 | **0.377** |
| 21 - fantasizing | 0.078 | -0.044 | **0.423** | 0.061 | 0.058 | **0.378** | **0.425** | 0.038 | 0.180 | **0.359** | -0.015 | **0.376** | 0.118 | -0.012 | **0.455** |
| 22 - preoccupied | 0.023 | **0.433** | 0.276 | 0.080 | **0.361** | 0.081 | 0.085 | 0.172 | 0.229 | 0.007 | **0.661** | 0.278 | -0.036 | **0.413** | 0.115 |
| 23 - defiant | **0.569** | -0.049 | 0.000 | 0.057 | **0.363** | -0.160 | 0.094 | -0.016 | **0.407** | 0.104 | **0.761** | 0.024 | 0.056 | 0.245 | 0.048 |
| 24 - pensive | 0.095 | 0.153 | **0.360** | 0.052 | **0.379** | 0.213 | 0.024 | **0.362** | **0.323** | **0.307** | **0.414** | 0.211 | -0.023 | -0.106 | **0.561** |
| 25 - interested | 0.148 | -0.144 | **0.427** | 0.176 | -0.078 | 0.266 | **0.344** | 0.275 | -0.270 | **0.317** | 0.040 | **0.378** | 0.220 | -0.107 | **0.305** |
| 26 - hostile | 0.265 | 0.009 | 0.027 | 0.032 | **0.354** | 0.085 | 0.134 | 0.271 | **0.377** | 0.041 | **0.441** | 0.133 | **0.399** | 0.257 | 0.189 |
| 27 – cautious | 0.057 | **0.545** | 0.140 | 0.147 | **0.384** | -0.007 | 0.020 | 0.250 | **0.363** | 0.274 | **0.732** | 0.019 | -0.145 | **0.427** | -0.014 |
| 28 - interested | 0.188 | 0.204 | 0.160 | 0.015 | **0.444** | 0.026 | 0.011 | 0.266 | **0.478** | 0.002 | **0.481** | 0.087 | 0.209 | **0.516** | 0.001 |
| 29 - reflective | 0.174 | -0.055 | **0.534** | 0.130 | 0.042 | 0.178 | 0.257 | -0.042 | -0.018 | 0.195 | 0.262 | **0.446** | 0.088 | -0.004 | 0.158 |
| 30 - flirtatious | 0.031 | **0.304** | **0.649** | 0.187 | 0.190 | **0.309** | **0.391** | **0.403** | 0.010 | **0.529** | 0.252 | 0.297 | -0.234 | 0.041 | **0.494** |
| 31 - confident | 0.155 | 0.119 | **0.366** | 0.114 | 0.000 | 0.290 | **0.333** | 0.019 | -0.034 | 0.114 | 0.057 | **0.517** | -0.015 | -0.069 | **0.469** |
| 32 - serious | 0.030 | **0.319** | 0.179 | 0.077 | 0.203 | 0.289 | 0.136 | 0.168 | 0.068 | 0.070 | **0.433** | 0.201 | 0.037 | -0.045 | **0.464** |
| 33 - concerned | 0.104 | 0.185 | 0.182 | 0.131 | 0.104 | 0.133 | 0.147 | 0.007 | 0.199 | **0.512** | 0.170 | 0.016 | 0.042 | 0.148 | 0.083 |
| 34 - distrustful | 0.207 | **0.340** | 0.098 | 0.059 | **0.380** | -0.011 | 0.089 | 0.114 | 0.281 | 0.076 | **0.560** | 0.027 | -0.202 | **0.472** | -0.017 |
| 35 - nervous | 0.024 | **0.449** | -0.080 | 0.066 | **0.432** | -0.193 | 0.047 | -0.025 | **0.456** | -0.23 | **0.738** | 0.149 | -0.132 | **0.398** | 0.056 |
| 36 - suspicious | 0.169 | -0.042 | 0.279 | 0.055 | **0.351** | 0.073 | 0.018 | 0.013 | **0.485** | **0.541** | **0.340** | 0.018 | -0.044 | 0.290 | 0.058 |
| Proportion variance | .053 | .074 | .103 | .030 | .108 | .039 | .060 | .048 | .099 | .121 | .213 | .067 | .040 | .085 | .074 |
| Total cumulative variance |  | .230 |  |  | .177 |  |  | .207 |  |  | .401 |  |  | .199 |  |
| Items with no loading > .3 |  | 12 |  |  | 7 |  |  | 10 |  |  | 1 |  |  | 7 |  |

*Note.* Factor loadings for EFA model in the datasets where the scree plot was suggestive of a multidimensional factor structure. f1 = factor 1. f2 = factor 2. f3 = factor 3. These factor names were assigned by *lavaan*, and f1 in one dataset does not necessary correspond to f1 in other datasets. We could not identify any consistent patterns of factor across datasets. Factor loadings > .30 are presented in bold font.

**Table S8**

*Standardised Factor Loadings for Three-factor EFA Models with Oblimin Rotation*

|  | Almaatouq | |  | Dodell-Feder | |  | Nahal |  |  | Panero | |  | Pearce |  |  |
| --- | --- | --- | --- | --- | --- | --- | --- | --- | --- | --- | --- | --- | --- | --- | --- |
| RMET item | f1 | f2 | f3 | f1 | f2 | f3 | f1 | f2 | f3 | f1 | f2 | f3 | f1 | f2 | f3 |
| 1 - playful | 0.040 | 0.018 | 0.274 | **0.606** | 0.008 | 0.013 | 0.047 | **-0.308** | **0.355** | 0.230 | 0.103 | **0.348** | 0.014 | 0.144 | 0.098 |
| 2 - upset | 0.145 | **0.602** | -0.026 | -0.012 | 0.174 | 0.103 | 0.168 | 0.021 | 0.163 | -0.084 | **0.376** | 0.218 | 0.078 | **0.527** | 0.079 |
| 3 - desire | 0.068 | -0.058 | **0.410** | 0.025 | 0.147 | **0.381** | **0.621** | 0.043 | -0.087 | **0.624** | -0.197 | 0.217 | **0.710** | 0.166 | -0.016 |
| 4 - insisting | **0.568** | -0.042 | 0.058 | -0.167 | **0.382** | -0.112 | 0.096 | **0.359** | 0.009 | **0.588** | 0.120 | **-0.380** | **0.476** | -0.182 | 0.060 |
| 5 - worried | 0.299 | 0.257 | -0.007 | -0.015 | 0.250 | 0.072 | 0.124 | 0.127 | 0.141 | **0.686** | 0.143 | -0.043 | 0.066 | 0.004 | **0.442** |
| 6 - fantasizing | 0.175 | -0.031 | **0.657** | **0.318** | -0.022 | **0.301** | **0.424** | -0.156 | 0.242 | **0.557** | -0.187 | 0.275 | 0.131 | 0.182 | -0.110 |
| 7 - uneasy | **0.339** | 0.280 | -0.153 | -0.272 | **0.399** | -0.019 | 0.135 | **0.491** | 0.012 | 0.219 | **0.453** | -0.214 | 0.116 | **-0.451** | 0.283 |
| 8 - despondent | **0.363** | 0.291 | **0.309** | -0.007 | **0.327** | 0.184 | -0.066 | -0.046 | **0.487** | **0.563** | 0.134 | 0.044 | 0.133 | 0.117 | **0.347** |
| 9 - preoccupied | 0.067 | 0.034 | **0.572** | -0.015 | **0.302** | **0.315** | 0.113 | -0.013 | **0.476** | **0.683** | 0.102 | 0.078 | **-0.371** | 0.256 | **0.447** |
| 10 - cautious | 0.183 | 0.204 | 0.043 | -0.175 | **0.398** | -0.061 | -0.020 | 0.262 | 0.009 | **0.389** | 0.030 | -0.101 | 0.162 | -0.157 | 0.294 |
| 11 - regretful | **0.358** | 0.214 | -0.099 | 0.037 | **0.391** | -0.065 | 0.049 | **0.452** | 0.089 | **0.625** | 0.090 | 0.000 | 0.076 | 0.141 | **0.382** |
| 12 - sceptical | 0.233 | 0.158 | 0.112 | -0.042 | **0.436** | 0.046 | **0.409** | **0.354** | 0.045 | **0.348** | **0.370** | 0.011 | 0.129 | -0.155 | **0.435** |
| 13 - anticipating | 0.112 | 0.149 | 0.222 | 0.151 | **0.311** | 0.061 | **0.323** | 0.179 | 0.087 | **0.566** | 0.270 | -0.074 | -0.114 | -0.063 | **0.343** |
| 14 - accusing | **0.395** | -0.179 | **0.306** | -0.013 | **0.458** | 0.082 | **0.409** | 0.107 | 0.091 | 0.263 | **0.579** | -0.077 | 0.259 | 0.283 | 0.202 |
| 15 -contemplative | **0.329** | -0.020 | **0.339** | 0.078 | **0.454** | 0.119 | 0.201 | 0.209 | **0.312** | 0.207 | **0.387** | 0.128 | 0.071 | -0.094 | **0.485** |
| 16 - thoughtful | 0.036 | **0.306** | **0.393** | 0.092 | 0.214 | 0.151 | -0.113 | 0.004 | **0.401** | 0.167 | 0.266 | **0.388** | -0.010 | 0.064 | 0.208 |
| 17 - doubtful | **0.341** | 0.299 | -0.138 | 0.153 | **0.381** | **-0.376** | **-0.332** | **0.351** | 0.292 | 0.163 | **0.467** | -0.262 | -0.072 | -0.249 | **0.309** |
| 18 - decisive | 0.201 | 0.157 | 0.133 | -0.029 | **0.358** | 0.052 | 0.169 | 0.272 | 0.132 | **0.544** | -0.060 | -0.229 | 0.245 | 0.224 | 0.150 |
| 19 - tentative | 0.020 | 0.259 | 0.038 | -0.013 | **0.350** | 0.064 | 0.162 | 0.142 | **0.357** | -0.070 | **0.342** | 0.064 | -0.224 | 0.103 | **0.326** |
| 20 - friendly | 0.056 | 0.294 | 0.204 | 0.171 | 0.140 | **0.411** | 0.016 | -0.230 | **0.455** | 0.099 | **0.418** | **0.538** | 0.117 | **0.338** | 0.294 |
| 21 - fantasizing | 0.159 | -0.080 | **0.393** | -0.024 | 0.045 | **0.414** | **0.437** | 0.095 | 0.127 | **0.405** | -0.144 | **0.347** | 0.223 | **0.377** | 0.016 |
| 22 - preoccupied | 0.023 | **0.421** | 0.248 | 0.089 | **0.331** | 0.150 | 0.069 | 0.063 | 0.297 | 0.186 | **0.506** | **0.326** | 0.041 | 0.098 | **0.422** |
| 23 - defiant | **0.595** | -0.079 | -0.122 | 0.123 | **0.339** | -0.105 | 0.135 | 0.292 | 0.196 | 0.093 | **0.637** | 0.036 | 0.094 | 0.010 | 0.241 |
| 24 - pensive | 0.164 | 0.125 | **0.318** | -0.060 | **0.347** | 0.256 | -0.073 | 0.026 | **0.545** | **0.471** | 0.222 | 0.213 | 0.103 | **0.535** | -0.055 |
| 25 - interested | 0.076 | -0.170 | **0.451** | 0.094 | -0.081 | **0.309** | 0.262 | **-0.349** | 0.136 | **0.371** | -0.088 | **0.356** | 0.273 | 0.204 | -0.100 |
| 26 - hostile | 0.282 | -0.006 | -0.032 | -0.015 | **0.327** | 0.126 | 0.111 | 0.111 | **0.477** | 0.072 | **0.355** | 0.169 | **0.458** | 0.007 | 0.233 |
| 27 - cautious | 0.081 | **0.541** | 0.094 | 0.173 | **0.353** | 0.078 | -0.041 | 0.116 | **0.450** | -0.115 | **0.664** | 0.095 | -0.094 | 0.020 | **0.434** |
| 28 - interested | 0.224 | 0.186 | 0.102 | 0.050 | **0.410** | 0.088 | -0.025 | 0.187 | **0.528** | 0.135 | **0.376** | 0.121 | 0.259 | -0.108 | **0.492** |
| 29 - reflective | 0.084 | -0.086 | **0.555** | 0.083 | 0.032 | 0.222 | 0.266 | 0.005 | -0.058 | 0.288 | 0.121 | **0.452** | 0.123 | 0.113 | 0.001 |
| 30 - flirtatious | 0.150 | 0.259 | **0.602** | 0.119 | 0.166 | **0.390** | **0.308** | -0.225 | **0.415** | **0.683** | 0.020 | 0.268 | -0.099 | **0.547** | 0.104 |
| 31 - confident | 0.095 | 0.102 | **0.379** | 0.039 | -0.008 | **0.328** | **0.328** | -0.042 | -0.005 | 0.132 | -0.021 | **0.518** | 0.092 | **0.444** | -0.027 |
| 32 - serious | 0.001 | **0.312** | 0.162 | 0.025 | 0.181 | **0.344** | 0.107 | -0.048 | 0.207 | 0.197 | **0.310** | 0.227 | 0.144 | **0.419** | -0.009 |
| 33 - concerned | 0.141 | 0.169 | 0.143 | 0.100 | 0.091 | 0.183 | 0.165 | 0.132 | 0.109 | **0.650** | -0.023 | -0.022 | 0.077 | 0.053 | 0.150 |
| 34 - distrustful | 0.232 | **0.328** | 0.031 | 0.093 | **0.351** | 0.052 | 0.091 | 0.132 | 0.264 | 0.247 | **0.420** | 0.058 | -0.144 | 0.037 | **0.484** |
| 35 - nervous | 0.043 | **0.464** | -0.095 | 0.145 | **0.403** | -0.128 | 0.000 | **0.334** | 0.216 | -0.057 | **0.666** | -0.080 | -0.068 | 0.082 | **0.411** |
| 36 - suspicious | 0.228 | -0.072 | 0.234 | -0.033 | **0.325** | 0.107 | 0.060 | **0.331** | 0.269 | **0.733** | 0.106 | -0.048 | 0.004 | 0.055 | 0.296 |
| Proportion variance | .066 | .071 | .093 | .026 | .098 | .053 | .060 | .055 | .092 | .198 | .133 | .070 | .049 | .065 | .086 |
| Total cumulative variance |  | .230 |  |  | .177 |  |  | .207 |  |  | .401 |  |  | .199 |  |
| Items with no loading > .3 |  | 11 |  |  | 4 |  |  | 10 |  |  | 0 |  |  | 11 |  |

*Note.* Factor loadings for EFA model in the datasets where the scree plot was suggestive of a multidimensional factor structure. f1 = factor 1. f2 = factor 2. f3 = factor 3. These factor names were assigned by *lavaan*, and f1 in one dataset does not necessary correspond to f1 in other datasets. We could not identify any consistent patterns of factor across datasets. Factor loadings > .30 are presented in bold font.

**Table S9**

*Communalities for Two and Three-Factor EFA Models with Geomin Rotation (as Preregistered)*

| **Dataset** | **Almaatouq** | | | **Dodell** | | **Nahal** | | | **Panero** | | | **Pearce** | |
| --- | --- | --- | --- | --- | --- | --- | --- | --- | --- | --- | --- | --- | --- |
| Item - target | 2-Factor | 3-Factor | 2-Factor | | 3-Factor | 2-Factor | 3-Factor | 2-Factor | | 3-Factor | 2-Factor | | 3-Factor |
| R01 - playful | 0.073 | 0.074 | 0.106 | | 0.373 | 0.121 | 0.178 | 0.265 | | 0.278 | 0.032 | | 0.036 |
| R02 - upset | 0.127 | 0.317 | 0.049 | | 0.051 | 0.076 | 0.076 | 0.110 | | 0.169 | 0.288 | | 0.312 |
| R03 - desire | 0.165 | 0.173 | 0.195 | | 0.209 | 0.184 | 0.359 | 0.388 | | 0.419 | 0.217 | | 0.551 |
| R04 - insisting | 0.195 | 0.326 | 0.139 | | 0.142 | 0.121 | 0.144 | 0.270 | | 0.415 | 0.023 | | 0.249 |
| R05 - worried | 0.207 | 0.206 | 0.077 | | 0.077 | 0.073 | 0.075 | 0.536 | | 0.582 | 0.208 | | 0.206 |
| R06 - fantasizing | 0.400 | 0.398 | 0.210 | | 0.231 | 0.304 | 0.307 | 0.382 | | 0.396 | 0.054 | | 0.058 |
| R07 - uneasy | 0.230 | 0.230 | 0.143 | | 0.188 | 0.218 | 0.272 | 0.349 | | 0.353 | 0.181 | | 0.254 |
| R08 - despondent | 0.491 | 0.493 | 0.174 | | 0.178 | 0.135 | 0.210 | 0.425 | | 0.443 | 0.176 | | 0.176 |
| R09 - preoccupied | 0.362 | 0.365 | 0.233 | | 0.248 | 0.249 | 0.274 | 0.573 | | 0.603 | 0.155 | | 0.388 |
| R10 - cautious | 0.112 | 0.111 | 0.145 | | 0.155 | 0.065 | 0.069 | 0.114 | | 0.147 | 0.099 | | 0.127 |
| R11 - regretful | 0.201 | 0.206 | 0.144 | | 0.147 | 0.227 | 0.241 | 0.425 | | 0.463 | 0.195 | | 0.196 |
| R12 - sceptical | 0.138 | 0.140 | 0.199 | | 0.199 | 0.230 | 0.333 | 0.409 | | 0.409 | 0.201 | | 0.216 |
| R13 - anticipating | 0.127 | 0.127 | 0.147 | | 0.159 | 0.140 | 0.179 | 0.513 | | 0.539 | 0.109 | | 0.122 |
| R14 - accusing | 0.136 | 0.261 | 0.238 | | 0.238 | 0.174 | 0.223 | 0.561 | | 0.558 | 0.225 | | 0.230 |
| R15 - contemplative | 0.218 | 0.270 | 0.280 | | 0.280 | 0.263 | 0.263 | 0.323 | | 0.333 | 0.239 | | 0.240 |
| R16 - thoughtful | 0.318 | 0.335 | 0.112 | | 0.112 | 0.082 | 0.143 | 0.332 | | 0.374 | 0.048 | | 0.051 |
| R17 - doubtful | 0.253 | 0.247 | 0.119 | | 0.213 | 0.228 | 0.287 | 0.337 | | 0.338 | 0.140 | | 0.138 |
| R18 - decisive | 0.129 | 0.130 | 0.139 | | 0.139 | 0.146 | 0.159 | 0.124 | | 0.230 | 0.152 | | 0.160 |
| R19 - tentative | 0.061 | 0.079 | 0.139 | | 0.139 | 0.242 | 0.242 | 0.078 | | 0.101 | 0.081 | | 0.159 |
| R20 - friendly | 0.115 | 0.150 | 0.304 | | 0.298 | 0.140 | 0.213 | 0.492 | | 0.621 | 0.257 | | 0.258 |
| R21 - fantasizing | 0.164 | 0.189 | 0.149 | | 0.181 | 0.216 | 0.266 | 0.327 | | 0.324 | 0.221 | | 0.211 |
| R22 - preoccupied | 0.265 | 0.311 | 0.190 | | 0.19 | 0.116 | 0.121 | 0.533 | | 0.592 | 0.204 | | 0.206 |
| R23 - defiant | 0.149 | 0.313 | 0.105 | | 0.128 | 0.188 | 0.194 | 0.465 | | 0.493 | 0.070 | | 0.072 |
| R24 - pensive | 0.205 | 0.207 | 0.213 | | 0.232 | 0.199 | 0.282 | 0.520 | | 0.520 | 0.290 | | 0.300 |
| R25 - interested | 0.177 | 0.184 | 0.104 | | 0.105 | 0.204 | 0.199 | 0.321 | | 0.320 | 0.098 | | 0.126 |
| R26 - hostile | 0.048 | 0.075 | 0.144 | | 0.147 | 0.303 | 0.317 | 0.189 | | 0.214 | 0.149 | | 0.286 |
| R27 - cautious | 0.303 | 0.372 | 0.194 | | 0.209 | 0.192 | 0.230 | 0.301 | | 0.385 | 0.173 | | 0.192 |
| R28 - interested | 0.146 | 0.146 | 0.212 | | 0.211 | 0.310 | 0.353 | 0.242 | | 0.256 | 0.276 | | 0.324 |
| R29 - reflective | 0.279 | 0.276 | 0.073 | | 0.071 | 0.032 | 0.063 | 0.425 | | 0.450 | 0.028 | | 0.030 |
| R30 - flirtatious | 0.616 | 0.616 | 0.264 | | 0.266 | 0.346 | 0.353 | 0.676 | | 0.686 | 0.214 | | 0.325 |
| R31 - confident | 0.153 | 0.162 | 0.103 | | 0.113 | 0.076 | 0.107 | 0.283 | | 0.329 | 0.205 | | 0.210 |
| R32 - serious | 0.126 | 0.154 | 0.187 | | 0.198 | 0.065 | 0.067 | 0.291 | | 0.308 | 0.208 | | 0.206 |
| R33 - concerned | 0.110 | 0.109 | 0.075 | | 0.075 | 0.072 | 0.079 | 0.333 | | 0.396 | 0.036 | | 0.037 |
| R34 - distrustful | 0.223 | 0.224 | 0.157 | | 0.162 | 0.131 | 0.131 | 0.374 | | 0.376 | 0.203 | | 0.248 |
| R35 - nervous | 0.098 | 0.188 | 0.146 | | 0.181 | 0.197 | 0.194 | 0.366 | | 0.398 | 0.165 | | 0.184 |
| R36 - suspicious | 0.084 | 0.117 | 0.131 | | 0.135 | 0.246 | 0.244 | 0.552 | | 0.613 | 0.094 | | 0.096 |
